# Molecular Docking Simulation Studies Identifies Potential Natural Product Derived-Antiwolbachial Compounds as Filaricides against Onchocerciasis

**DOI:** 10.3390/biomedicines9111682

**Published:** 2021-11-13

**Authors:** Samuel K. Kwofie, Emmanuel Broni, Faruk U. Yunus, John Nsoh, Dela Adoboe, Whelton A. Miller, Michael D. Wilson

**Affiliations:** 1 Department of Biomedical Engineering, School of Engineering Sciences, College of Basic and Applied Sciences, University of Ghana, PMB LG 77, Legon, Accra LG 77, Ghana; ebroni002@st.ug.edu.gh (E.B.); farukyunus.fy@gmail.com (F.U.Y.); nsoh.john@gmail.com (J.N.); justadoboedela@yahoo.co.uk (D.A.); 2 West African Centre for Cell Biology of Infectious Pathogens, Department of Biochemistry, Cell and Molecular Biology, College of Basic and Applied Sciences, University of Ghana, Accra LG 54, Ghana; 3 Department of Parasitology, Noguchi Memorial Institute for Medical Research (NMIMR), College of Health Sciences (CHS), University of Ghana, P.O. Box LG 581, Legon, Accra LG 581, Ghana; MWilson@noguchi.ug.edu.gh; 4 Department of Medicine, Loyola University Medical Center, Maywood, IL 60153, USA; wmiller6@luc.edu; 5 Department of Molecular Pharmacology and Neuroscience, Loyola University Medical Center, Maywood, IL 60153, USA; 6 Department of Chemical and Biomolecular Engineering, School of Engineering and Applied Science, University of Pennsylvania, Philadelphia, IL 19104, USA

**Keywords:** onchocerciasis, *Onchocerca volvulus*, *Wolbachia* surface protein, structure-based drug design, natural product

## Abstract

Onchocerciasis is the leading cause of blindness and severe skin lesions which remain a major public health problem, especially in tropical areas. The widespread use of antibiotics and the long duration required for effective treatment continues to add to the increasing global menace of multi-resistant pathogens. *Onchocerca volvulus* harbors the endosymbiont bacteria *Wolbachia*, essential for the normal development of embryos, larvae and long-term survival of the adult worm, *O. volvulus*. We report here results of using structure-based drug design (SBDD) approach aimed at identifying potential novel *Wolbachia* inhibitors from natural products against the *Wolbachia* surface protein (WSP). The protein sequence of the WSP with UniProtKB identifier Q0RAI4 was used to model the three-dimensional (3D) structure via homology modelling techniques using three different structure-building algorithms implemented in Modeller, I-TASSER and Robetta. Out of the 15 generated models of WSP, one was selected as the most reasonable quality model which had 82, 15.5, 1.9 and 0.5% of the amino acid residues in the most favored regions, additionally allowed regions, generously allowed regions and disallowed regions, respectively, based on the Ramachandran plot. High throughput virtual screening was performed via Autodock Vina with a library comprising 42,883 natural products from African and Chinese databases, including 23 identified anti-*Onchocerca* inhibitors. The top six compounds comprising ZINC000095913861, ZINC000095486235, ZINC000035941652, NANPDB4566, acetylaleuritolic acid and rhemannic acid had binding energies of −12.7, −11.1, −11.0, −11, −10.3 and −9.5 kcal/mol, respectively. Molecular dynamics simulations including molecular mechanics Poisson-Boltzmann (MMPBSA) calculations reinforced the stability of the ligand-WSP complexes and plausible binding mechanisms. The residues Arg45, Tyr135, Tyr148 and Phe195 were predicted as potential novel critical residues required for ligand binding in pocket 1. Acetylaleuritolic acid and rhemannic acid (lantedene A) have previously been shown to possess anti-onchocercal activity. This warrants the need to evaluate the anti-WSP activity of the identified molecules. The study suggests the exploitation of compounds which target both pockets 1 and 2, by investigating their potential for effective depletion of *Wolbachia*. These compounds were predicted to possess reasonably good pharmacological profiles with insignificant toxicity and as drug-like. The compounds were computed to possess biological activity including antibacterial, antiparasitic, anthelmintic and anti-rickettsials. The six natural products are potential novel antiwolbachial agents with insignificant toxicities which can be explored further as filaricides for onchocerciasis.

## 1. Introduction

Onchocerciasis, caused by *Onchocerca volvulus* is a major cause of blindness and skin lesions in the tropics, notably in sub-Saharan African countries, Yemen, and in specific areas in the Americas; Brazil, Mexico, Guatemala, Colombia, Venezuela and Ecuador [1,2,3]. The World Health Organization (WHO) estimates that about 37 million people are infected [4,5] with about 600,000 blind, and 500,000 Africans visually impaired [6]. Onchocerciasis-associated epilepsy cases have been reported although studies have shown that *O. volvulus* microfilariae and *Wolbachia* are not present in the brain parenchyma and cerebrospinal fluid [7,8]. The disease has socio-economic consequences, causing infected persons to lose their productivity, thereby, negatively impacting on the rural economies of afflicted communities.

Ivermectin (IVM) which is an effective microfilaricide is the recommended drug for treating onchocerciasis [9] and the control strategy is implemented through mass drug administration (MDA) programs which could be on a large scale. For example, in 2014 alone, more than 112 million people were treated in 22 African countries by the African Programme for Onchocerciasis Control (APOC) [10]. However, IVM has only a transient sterilizing effect on the adult worm [11], hence, a long duration of therapy would be required to have marked effect on the adult female *O. volvulus* [12,13] which can live up to 15 years [14].

The large-scale use of IVM can only increase drug pressure on the parasite, increasing the risk of development of resistance. Under-dosing resulting from MDA strategy of single-dose administration of anthelmintics, irrespective of the intensities of infection can also be another reason for progressively selecting for more drug-tolerant parasites. Already low respondents to IVM, evidenced by suboptimal efficacy in endemic communities have been reported [15,16,17,18], which adds credence to this possibility [16,19,20]. Few drugs including moxidectin, emodepside, flubendazole and tribendimidine are currently available and are undergoing various stages of trial as possible alternatives to IVM [21,22]. The aforementioned are all developments from veterinary drugs and are broad-spectrum anthelmintic except emodepside which has a novel mechanism of action, thus cross-resistance between them are likely [21,23].

Like most filarial nematodes, *O. volvulus* also harbors *Wolbachia*, an intracellular bacterial symbiont throughout its life cycle [24,25] and are important for the normal development of embryos, larvae and long-term survival of the adult *O. volvulus. Wolbachia* released from the worms are also believed to be involved in the pathogenesis of onchocerciasis [26,27,28,29].

*Wolbachia* surface protein (WSP), an abundantly expressed protein of *Wolbachia* [30,31], appears to be highly conserved in *Wolbachia* from filarial nematodes and has been used for investigating the endosymbiont phylogeny [32,33]. Filarial infection is associated with Th2 cell responses thereby activating potentially toxic effectors such as eosinophils [34,35]. A *Wolbachia*-derived distinct protein (WSP) can elicit in vitro inflammatory responses [36]. The WSP of *Brugia malayi* (wBm00432) is in complex with 6 *B. malayi* glycolytic enzymes, including aldolase [37,38]. WSP family proteins have been suggested to play crucial roles in anchoring the *Wolbachia* to *B. malayi*’s cytoskeleton, as well as optimizing the energy production pathway in the parasite [37]. Surface proteins encoded by bacteria are very important in bacterial pathogenesis [39]. Bacterial surface proteins are involved in interactions with the cell environment and can also be involved in adhesion and invasion of the host cells, as well as in defending against host responses, which makes them potential drug targets [40,41].

Therefore, the proteins in *Wolbachia* bacteria have become important targets for antifilarial therapy [27,42] and antibiotics-based treatment regimens with doxycycline (DOX), tetracycline (TET) and its derivatives [43,44]. The advantages which have stimulated the development of these new drugs are the slow depletion of *Wolbachia* that avoids inflammatory reactions associated with IVM and diethylcarbamazine (DEC) treatments [45], and also can safely be used in loiasis co-endemic areas because *Loa loa* does not contain *Wolbachia* [46].

The standard recommended use of DOX is 200 mg/day for 4 weeks [47], which is a long treatment for an antibiotic use, likewise for minocycline 200 mg/day for 3 weeks. The use of antibiotics over long periods of time to treat neglected tropical diseases is not limited to filarial diseases alone, combination therapy of streptomycin (15 mg/kg body weight daily) and rifampicin (10 mg/kg (8–12 mg/kg) daily, maximum 600 mg daily) for 8 weeks is used in Buruli ulcer disease. The overuse and abuse of antibiotics are the major contributory factors to the global crisis of antibiotic resistance [48], with multi-drug resistance developing in common bacterial pathogens, aggravating post-surgery management and tuberculosis treatment.

A search on Protein Data Bank as at 28 May 2021 revealed that only five solved proteins pertaining to *Onchocerca volvulus* with PDB IDs 1TU7, 2HNL, 6MN8, 1TU8 and 5IN2 were available. Additionally, due to the absence of a crystallographic structure of *O. volvulus* chitinase (OvCHT1), a previous study developed a homology model of (OvCHT1) using the available X-ray structures of human chitinases as templates [49]. It has therefore become imperative to model the structure of the *Wolbachia* surface protein due to the paucity of structural data pertaining to receptors of the *O. volvulus*.

The lack of vaccine and reasonably safe macrofilaricidal treatment against *O. volvulus* necessitate the need to explore natural products to unravel new lead molecules [50]. There is need to find novel antiwolbachial leads with therapeutic potential against onchocerciasis [51]. Major sources of such compounds are plants products which possess enormous structural and chemical diversity. Furthermore, they have the additional advantage of generating compounds with structural and physiochemical properties not similar to the existing synthetic therapeutic agents [52,53].

This study, therefore, sought to identify potential novel antiwolbachial compounds from freely available natural products. To achieve this, small compounds were virtually screened against the WSP of *O. volvulus*. Further in silico analysis was performed to elucidate the mechanisms of binding and pharmacological profiles of the screened compounds. The biological activity of selected compounds was also determined using the Prediction of Activity Spectra of Substances (PASS), a Bayesian-based model predicting the probable activity of small molecules [54,55,56].

## 2. Materials and Methods

**Figure 1:**
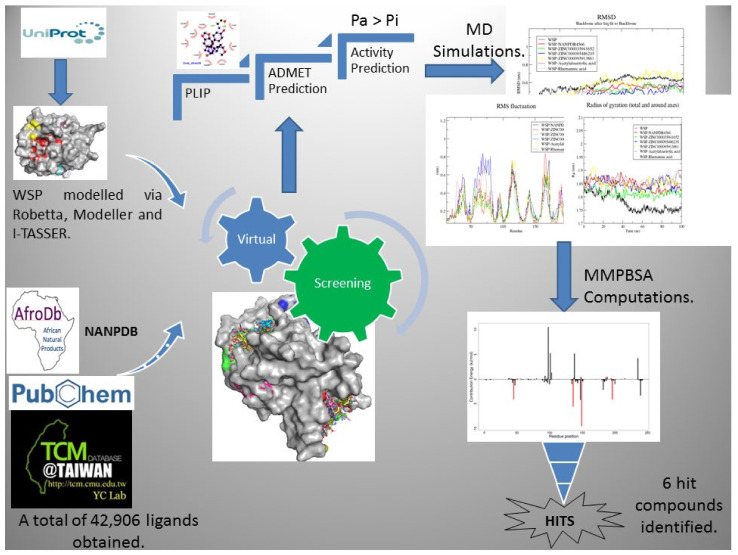
Methodology schema employed in this study to predict potential antiwolbachial biomolecules. Three different modelling approaches comprising Modeller, I-TASSER and Robetta were used to predict possible structures. Quality evaluations of the structures revealed the reasonably best model. Natural products-derived compounds from AfroDB, NANPDB, TCM and literature were used as screening library. The top performing compounds were subjected to ADME prediction and MD simulations. The study further predicts the biological activity of the selected biomolecules.

A schematic workflow of the step-by-step techniques used in the study is shown in Figure 1. A reasonably good structure of the WSP was modelled and validated, after which structure-based virtual screening (SBVS) was performed in order to identify compounds with high binding affinity to the WSP. MD simulations including MMPBSA computations were performed on the WSP-ligand complexes to determine the molecular interactions and the stability during binding. Absorption, distribution, metabolism, excretion and toxicity (ADMET) predictions were performed to evaluate the pharmacological profiles of the selected compounds. Furthermore, the biological activity of identified compounds was predicted using a machine learning-based technique [54,55].

### 2.1. Sequence Retrieval

The structure of the *Wolbachia* surface protein (WSP) of *O. volvulus* has not been experimentally solved therefore a 3D structure was built using homology modelling. The primary sequence of WSP was retrieved from Universal Protein Resource (UniProt) with ID Q0RAI4. Basic Local Alignment Search Tool (BLAST) search with default parameters was performed using SWISS MODEL in order to obtain suitably identical templates to the WSP [57,58].

### 2.2. Prediction of the Protein Structures

Since there was no experimentally solved structure of the WSP in the PDB, it warranted the need to model a reasonably quality model. Comparative studies of various homology modelling techniques have shown that no particular technique is superior in all aspects of quality than the others [59,60,61]. The family of the protein and the percentage sequence identity between the query and the template structure may influence the quality of a modelled structure [60]. Most of the modelling algorithms have been shown to generate high quality models when the sequence identity between the query and the template is greater than 35% [60,61]. However, as the sequence identity reduces, it becomes extremely difficult for the various modelling algorithms to provide high quality structures [60]. It is therefore necessary to employ different modelling techniques when building protein structures which have low sequence identities with their templates [62]. The reasonably suitable model can be chosen after the modelled structures are subjected to various quality evaluations.

Due to the low sequence identities of the retrieved templates, homology modelling using 3D structures from Protein Data Bank (PDB) was employed. Modeller 9.2 embedded in EasyModeller 4.0 [63] was used to model five possible structures of the WSP based on the three selected templates. Additionally, the iterative threading assembly refinement (I-TASSER) and Robetta were employed to predict plausible structures of the WSP [64,65,66,67,68,69]. 

The main and side chain conformations of the models were then predicted and corrected using both Scwrl4 and ModRefiner [70,71,72]. The reasonably best models from each of the 3 approaches were evaluated in order to select the most suitable model.

### 2.3. Model Quality Assessment

The quality of the modelled structures were assessed using SAVES v5.0 (https://servicesn.mbi.ucla.edu/SAVES/, accessed on 5 October 2019), a metaserver that runs six programs for checking and validating protein structures. The programs ran by SAVES v5.0 employed in this study include ERRAT [73], PROVE [74], VERIFY [75,76] and PROCHECK [77]. Additionally, ProSA-web was used to evaluate the model quality. Ramachandra plots were generated using PROCHECK [77]. The model with the best quality parameters from all the evaluations was selected as the most reasonable model of WSP for downstream analysis.

### 2.4. Obtaining Compounds

Ligand structures were retrieved from AfroDB, North African Natural Products Database (NANPDB) and TCM@Taiwan. A total of **880** compounds were retrieved from AfroDB, a catalog of the ZINC15 database containing naturally occurring African compounds [78,79]. A total of **6842** compounds were also retrieved from NANPDB, a library of compounds isolated mainly from plants, with contributions from some endophyte, animal, fungal, and bacterial sources [80]. Additionally, a total of 35,161 traditional Chinese medicine (TCM) natural products were obtained from the world’s largest non-commercial TCM database [81], TCM@Taiwan, a catalog of the ZINC15 database. In all, a total of 42,883 ligands were obtained and used in this study, with **7722** compounds from the African flora. The unique identifiers (IDs) of the NANPDB compounds have been concatenated with the prefix “NANPDB” in this study.

Compounds which have been experimentally shown to possess inhibitory properties against other *Onchocerca* species were curated. The aim was to perform molecular docking studies with these compounds and examine the likelihood of repurposing them as WSP inhibitory compounds for the treatment of Onchocerciasis. A total of **23** compounds were found in literature as either anti-onchocercal compounds or major constituents of anti-*Onchocerca* plant extracts.

### 2.5. Prediction of Binding Sites

Computed Atlas of Structure Surface Topography of proteins (CASTp) was employed to predict potential ligand binding sites of the WSP protein [82,83]. The results were visually inspected using Chimera 1.12 (University of California San Francisco, version 1.12, San Francisco, CA, USA) and PyMOL 1.7.4.5 (PyMOL Molecular Graphics System, version 1.7.4.5, Schrödinger, LLC, New York, NY, USA). The area and volumes of the predicted sites were taken into consideration when selecting the plausible binding cavities.

### 2.6. Preparation of Protein and Ligands

The NANPDB and TCM compounds were converted from the simplified molecular input line entry system (SMILES) format to the Structure Data File (SDF) format. The natural products were divided into two categories: the African natural products (Afro) and the traditional Chinese natural products (TCM) comprising **7722** and **35,161** compounds, respectively. OSIRIS DataWarrior (Idorsia Pharmaceuticals Ltd., version 5.0.0, Allschwil, Switzerland) was used to filter the compound libraries by eliminating ligands with molecular weights greater than 600 g/mol and less than 150 g/mol [84]. Duplicates were also removed from the TCM library using DataWarrior. All ligand structures were energy minimized using the mmff94 force field and conjugate gradient algorithm in 200 steps using Open Babel [85,86]. The compounds were then converted to AutoDock Vina’s compatible Protein Data Bank partial charge and atom type format (PDBQT) [85,87]. 

Energy minimization of the 3 top WSP models (ROB2, ITAS1 and MOD2) were performed with GROMACS2018 using the Optimized Potentials for Liquid Simulations (OPLS)/All Atom (AA) force field. The refined and energy minimized protein structures were checked for the presence of water in order to prevent any post docking simulation interference by water. The protein structures were then saved in the Protein Data Bank (pdb) format using PyMOL 1.7.4.5 [88,89]. The protein structures were then converted to AutoDock Vina’s compatible pdbqt format using the “make macromolecule” option in PyRx prior to the molecular docking studies.

### 2.7. Molecular Docking

Autodock Vina was used to perform the docking simulations. The molecular docking simulations were performed in 2 stages. In the first stage, the obtained compounds were screened against the ROB2 model. The grid map was placed around the ROB2 protein structure with size dimensions of 46.8135 × 44.5267 × 61.2184 Å3 (for size_x, size_y and size_z, respectively) and center: 56.9654, 31.5760 and 39.6027 Å (for center_x, center_y and center_z, respectively). This grid map was used so as to investigate the presence of other binding sites which might not have been predicted by CASTp. AutoDock Vina, which uses the Lamarckian algorithm [81] was employed to search for eight different conformers for each ligand. All protein–ligand binding affinities are expressed as binding energy in kcal/mol. Analysis of the screening results was performed based on the three classifications: African natural products, TCM dataset and anti-*Onchocerca* spp. compounds. The top 10 percent of the African dataset was selected for downstream analysis. Additionally, the top 1 percent with high binding affinity from the TCM screening library was selected. The results of the molecular docking were visually inspected using Chimera and PyMOL 1.7.4.5 [87,88]. 

The second stage of molecular docking involved the screening of the top **20** compounds each of the Afro and TCM libraries against the ITAS1 and MOD2 protein structures. The **11** shortlisted Onchocerca spp. related compounds were also screened against both structures. This stage was carried out to identify compounds that bind to all three structures. Since the structures were modelled with templates of low sequence identities, multiple docking against the various comparative models could lead to substantial enrichment of the lead compounds [90].

### 2.8. ADMET Screening of Ligands

Ligands with high binding affinity and docked deep into the WSP model were subjected to absorption, distribution, metabolism and excretion (ADME) profiling using Swiss ADME and OSIRIS DataWarrioir 5.0.0 [91]. Swiss ADME was also used to determine the drug-likeness of the compounds based on Lipinski’s and Veber’s rules. The toxicity profiles of the hits were evaluated using the OSIRIS Property Explorer in DataWarrior. Relevant drug properties such as mutagenicity, tumorigenicity, irritancy and reproductive effect were predicted using the OSIRIS Property Explorer algorithm.

### 2.9. Characterisation of Binding Mechanisms

LigPLOT+ v1.4.5 [92] was employed to predict the protein–ligand interactions using default parameters. LigPLOT+ represents the protein–ligand interactions using 2D schematic diagrams where hydrogen bonds are shown as dashed green lines and hydrophobic interaction as arcs with spokes radiating towards the ligands.

### 2.10. Prediction of Biological Activities of Compounds

The biological activities of the shortlisted compounds were predicted using the Prediction of Activity Spectra for Substances (PASS) [54,56]. PASS predicts the biological activity spectra of compounds using the SMILES files of the structures present in its database via a Bayesian approach [54,56]. 

### 2.11. Molecular Dynamics Simulation

#### 2.11.1. Protein MD Simulation

The OPLS-AA/L all-atom force field was used to prepare the protein. To solvate the protein, it was centered in a cubic box, and placed at least 1.0 nm from the box edge. Then five sodium atoms were added to the solvent to neutralize the complex system. The solvated and electroneutral system was optimized through energy minimization using the steepest descent method. After minimization, 100 ps NVT (constant number of particles, volume and temperature) equilibration was conducted to stabilize the system. Then 100 ps NPT equilibration was followed to stabilize the pressure. Subsequent MD simulation of the protein was performed for 100 ns of constant pressure equilibration without constraints to relax the system.

#### 2.11.2. Protein–Ligand Complex MD Simulations

The topologies of the ligands were generated via CGenFF using default parameters prior to molecular dynamics (MD) simulation. GROMACS 2018 was employed to conduct the protein–ligand complex MD simulations under the CHARMM36 force field. In order to solvate each protein–ligand complex, the box boundaries were set to 1.0 nm away from the complexes. The charges of the complexes were also neutralized with sodium and chloride ions. Each complex was energy minimized and the final structures were used as inputs for the MD simulations. Additionally, equilibration protocol was used to restrain and relax protein–ligand positions. MD simulations for 100 ns were conducted with time steps of 2 fs under PME for each complex. The graphs generated from the MD simulations were plotted using Xmgrace.

#### 2.11.3. MM-PBSA Calculations of Ligand-Receptor Complexes

The binding free energies of the WSP-ligand complexes were investigated using the molecular mechanics Poisson-Boltzmann surface area (MMPBSA) method [93,94,95]. MMPBSA provides the combination of molecular mechanics and continuum solvent models. MMPBSA calculations of the complexes were carried out using the g_mmpbsa, which computes binding energy components and the individual energy contributions of the residues. The R programming package was then used to plot the MMPBSA graphs.

## 3. Results and Discussion

### 3.1. Primary Structure Analysis

The protein sequence of WSP is composed of 241 amino acid residues with UniProtKB ID: Q0RAI4. Determining the physiochemical parameters using ProtParam, the protein was predicted to have a molecular weight of 26,056.58 Da [96]. The WSP was also predicted to possess an instability index of 27.81 signifying the stable nature of the protein [96]. A protein is predicted to be stable when the instability index is lesser than 40 [96,97]. The chemical formula of WSP protein was C_1186_H_1813_N_289_O_357_S_7_.

### 3.2. Modelling of WSP Structure

#### 3.2.1. Template Identification

A BLAST search via SWISS-MODEL revealed a total of 223 templates to match the target sequence however, with relatively low sequence identities to the WSP. 1P4T was predicted to have the highest sequence identity to the query sequence (21.57%), and shared a similarity of 0.29 to the WSP (Table S1). A query via I-TASSER also indicated 1P4T as the best template with the highest sequence identity which corroborates the predictions by SWISS-MODEL. Additionally, both 2MLH and 2MAF showed sequence identity of 16.67% to the WSP (Table S1). The reasonably best template was selected based on the sequence identity, query coverage and the availability of a 3D structure.

#### 3.2.2. Structure Prediction Using Modeller

Modeller 9.2 was used to generate five possible model structures of the protein. The Neisserial surface protein A (NspA) from *Neisseria meningitidis* (PDB ID: 1P4T), which was solved using X-ray crystallography with a resolution of 2.55 Å [98], was selected as the main template for modelling the structure of the *Wolbachia* surface protein, but due to its low sequence identity (21.57%) (Table S1), multiple templates homology modelling using known 3D structures from the Protein Data Bank (PDB) was employed. The optimal use of several templates increases the model’s accuracy [99]. Two other templates, 2MAF and 2MLH, both with sequence identity of 16.67% were added to 1P4T for the multi-template modelling. The structures with PDB IDs 2MLH and 2MAF were solved using nuclear magnetic resonance (NMR) technique [100]. 1P4T, 2MAF and 2MLH belong to the same family of gram-negative bacteria outer membrane proteins and are therefore biologically related to the WSP. Five models were generated via Modeller and the qualities of these models were evaluated using the discrete optimized protein energy (DOPE) and genetic algorithm 341 (GA341) scores.

The GA341 score provides information on the reliability of a model, derived from statistical potentials [101]. A model is predicted to be reliable when the model score is higher than a pre-specified cut-off of 0.7. The five generated models had GA341 scores relatively lower than the cut-off. Thus, the reasonably best model was selected based on the DOPE score. DOPE is a statistical potential score used to assess homology models in protein structure prediction. The best model can be selected by picking the model with the lowest value of DOPE [102,103]. MOD2 was selected as the reasonably suitable model out of the five models generated using Modeller (Figure S1A and Table S2).

#### 3.2.3. Structure Prediction Using I-TASSER

I-TASSER was also employed to predict the model of the WSP. I-TASSER uses the SPICKER program to cluster all the decoys based on the pair-wise structure similarity, and reports five models which correspond to the five largest structure clusters [64,65,66,67]. The confidence of each model is quantitatively measured by C-score that is calculated based on the significance of threading template alignments and the convergence parameters of the structure assembly simulations. C-score is typically in the range of [−5, 2], where a C-score of a higher value signifies a model with a higher confidence and vice-versa [64,65,66,67]. The five models predicted by I-TASSER and their respective C-scores are shown (Table S3). For the I-TASSER predicted models, the model with the highest C-score, ITAS1 was selected as the reasonably suitable model (Figure S1B and Table S3).

#### 3.2.4. Structure Prediction Using Robetta

Robetta, which uses the ROSETTA algorithm also predicted five models using 1P4T as template, with 2K0L as the reference parent. 1P4T was predicted by Robetta as the most suitable template for modelling the structure of the protein. The structure 2K0L (KpOmpA) is an outer membrane protein A from *Klebsiella pneumoniae* transmembrane domain and was determined by NMR. KpOmpA, similar to the WSP, also induces specific humoral and cytotoxic responses, and is a potent carrier protein [104].

The predicted b-factors by color representation of the models were visualized in PyMOL 1.7.4.5. Generally, the b-factor which influences the local quality of a model shows the parts of the structure that were remodeled and not covered by a template. These are the least accurate regions and have the most variation between models. ROB1 and ROB2 showed similar b-factor coloration and had the least regions that were predicted without a template. The five models were then evaluated using SAVES v5.0 (Table S4). ROB2 had a VERIFY score of 70.95%, which was the highest; ERRAT quality factor of 78.97; PROVE score of 4.7% and two PROCHECK errors, three warnings and three passes (Table S4). ROB2 was therefore selected as the most acceptable structure from Robetta (Figure S1C).

#### 3.2.5. Model Quality Assessment

The quality of the three selected models from each technique was evaluated via SAVES v5.0 (Table S5). Protein structure MOD2 had a VERIFY score of 9.96%, ERRAT quality factor of 35.9649, PROVE score of 9.3% and three PROCHECK errors, two warnings and three passes (Table S5). Model ITAS1 was also predicted to have ERRAT, VERIFY and PROVE scores of 78.1116, 72.2% and 10%, respectively (Table S5). ITAS1 was also predicted to have seven PROCHECK errors, one warning and no pass. Model ROB2 was predicted to have a VERIFY score of 70.95%, ERRAT quality factor of 78.97, PROVE score of 4.7% and two PROCHECK errors, three warnings and two passes (Table S5). A high-quality model must have 80% of its amino acids score greater than or equal to 0.2 in the 3D-1D profile (VERIFY score). Although all the top three models did not have a VERIFY score of 80% or higher, ROB2 and ITAS 1 had relatively closer values. Additionally, a previous study has shown that this quality indicator (VERIFY) performed poorly on a crystallized structure [105]. The ERRAT plot of ROB2 showed that amino acid residues 147–150, 170, 172–178, 192–194, 205 and 209 were the most erroneous parts (Figure S2). 

The overall quality factor of the proteins as predicted by ERRAT showed that ROB2 was the highest with 78.97, followed by ITAS1 (78.1116) and MOD2 (35.9649). Although, the model ITAS1 had a higher VERIFY score of 72.2% as compared to ROB2 with 70.95%, ITAS1 was predicted by PROVE to be 10% erroneous while ROB2 was 4.7% erroneous. 

ModRefiner and Scwrl4 were used to repair the side chain conformations and refine the best models from each of the three techniques [70,71]. The Robetta and I-TASSER models showed no significant improvement in quality scores. However, the Modeller generated models demonstrated significant increase in their quality scores after using Scwrl4 and later refined using ModRefiner. After refining with Scwrl4 and ModRefiner, MOD2 had a verify score of 15.77%, ERRAT score of 54.5872, while PROCHECK reported five errors, one warning and two passes. 

**Figure 2:**
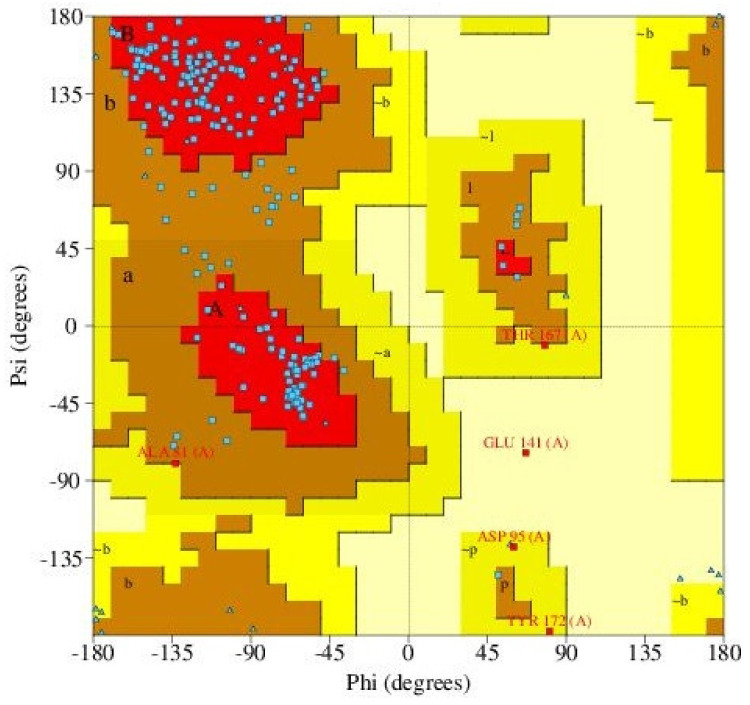
Ramachandran plot of the selected WSP model (ROB2) obtained via PROCHECK. The percentages of residues in the most favored regions, additionally allowed regions, generously allowed regions and disallowed regions are 82.0, 15.5, 1.9 and 0.5%, respectively.

The Ramachandran plots of all the three shortlisted models were generated using PROCHECK which evaluates the stereochemistry of protein models by determining the overall protein geometry and also, the residue-by-residue geometry [77] (Figure 2 and Figure S3A,B). The percentage of residues in the most favored, additionally allowed, generously allowed and disallowed regions determine the quality of protein structures [106]. WSP structure MOD2 had 82.5, 16.5, 1.0 and 0.0% of residues in the most favored, additionally allowed, generously allowed and disallowed regions, respectively (Table 1 and Figure S3A). ITAS1 was also predicted to have 53.9, 40.3, 3.9 and 1.9% of the residues in the most favored, additionally allowed, generously allowed and disallowed regions, respectively (Table 1 and Figure S3B). For the ROB2 structure, 82% of the amino acid residues were present in the most favored regions, 15.5% residues were found in the additionally allowed regions, 1.9% of the residues were in the generously allowed regions and 0.5% in the disallowed regions (Table 1 and Figure 2). 

**Figure 3:**
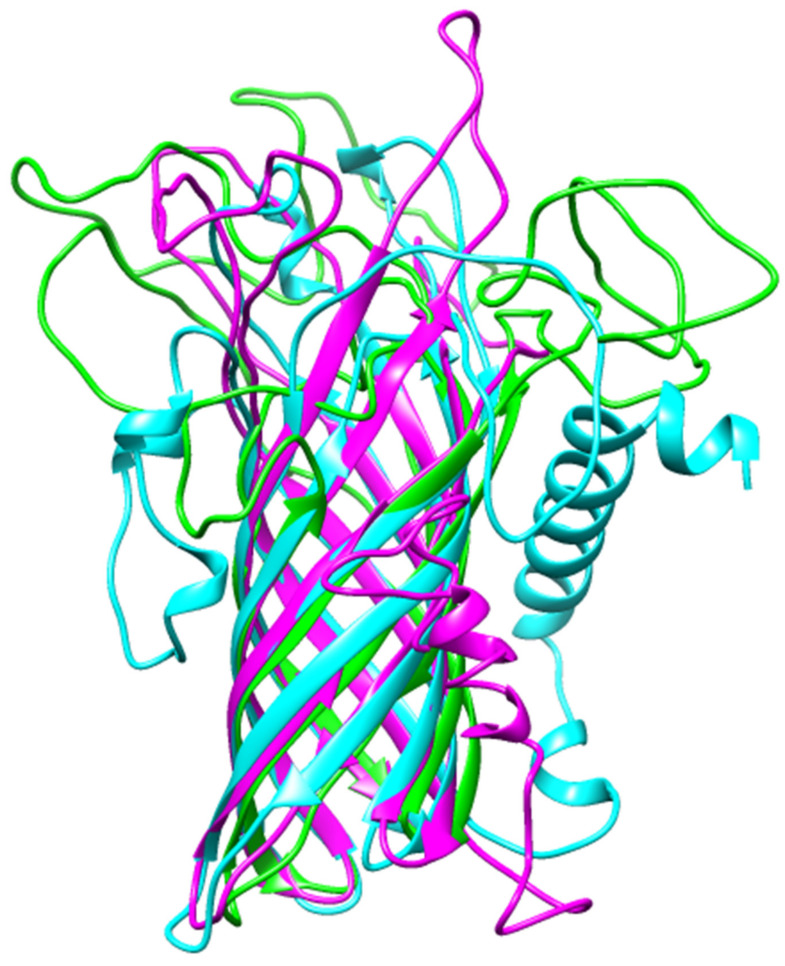
Alignment of the top three predicted tertiary structures of the *Wolbachia* surface protein from the 3 techniques using Chimera 1.12. ITAS1 is in magenta, ROB2 in cyan and MOD2 in green.

Structurally aligning all the three models using Chimera 1.12, it was observed that ITAS1 and ROB2 were structurally similar with an RMSD of 13.590 Å as compared to the alignments between MOD2 and ROB2 (RMSD of 26.529 Å); and ITAS1 and MOD2 (RMSD of 26.160 Å) (Figure 3). Considering the scores of the various quality evaluations (including the ERRAT, PROCHECK, VERIFY, PROVE scores and the Ramachandran plots) obtained for each model, ROB2 was selected as the reasonably accurate WSP model (Figure 2 and Figure S3A,B, Table 1 and Tables S2–S5).

#### 3.2.6. The Selected Model

Like NspA, the predicted WSP model forms β-barrels [98] consisting of eight β-strands with few alpha helices (Figure S1C). A comparative study of modelled WSPs of *Drosophila melanogaster*, *Asobera tabida* and *Brugia malayi* using the NspA as template reported that the WSP is an eight beta-barrel transmembrane structure [26]. 

The quality of the overall best WSP model (ROB2) was assessed using z-score from ProSA-web [107,108]. The z-score of the predicted WSP model is −4.02 and predicted to lie within the z-scores of NMR determined proteins (Figure S4A). The *z*-score indicates the overall model quality and is used to check whether the *z*-score of the input structure is within the range of scores typically found for native proteins of similar size [107,108]. The local model quality was also determined by plotting energies as a function of amino acid sequence position (Figure S4B). In general, positive values correspond to problematic or erroneous parts of the input structure [107,108]. Most of the residues of the WSP possessed relatively low energies signifying that the protein structure is less erroneous. It was observed that the residues towards the end of the sequence (after 160th position) had positive energy values and were erroneous, consistent with the ERRAT plot of the WSP (Figure S2).

### 3.3. Prediction of Binding Sites

CASTp predicted 40 binding sites for the WSP protein (model ROB2) [82,83,109]. CASTp provides information about the area, volume and the residues constituting the predicted binding site. Predicted binding sites with no openings and relatively small volumes and areas such that no ligand could fit were ignored [110,111]. Six binding sites were selected after visualizing using PyMOL 1.7.4.5 and Chimera 1.12 (Figure S5A,C). The residues lining each of the six binding sites are shown in Table 2. Of the six binding pockets, Pockets 1 and 2 were considered the most plausible sites due to their relatively large volumes and area (Table 2). The WSP (ROB2) was further aligned to the 1P4T template structure using ProBiS in order to identify conserved binding sites [112]. ProBiS detects conserved binding sites on a protein structure by performing a local, and surface oriented structural comparison of a query structure to a database of non-redundant protein structures [112]. ProBiS finds proteins that are locally similar to the query and calculates degrees of structural conservation for amino acid residues of the query protein [112]. The degrees of structural conservation are represented as colors, from blue to red signifying unconserved and conserved regions, respectively [112]. Pocket 1 was predicted by ProBiS as highly conserved among outer membrane proteins including 1P4T and 1QJP (Figure S5A,B) [98,113]. Pocket 1 is the “loop site” of outer membrane proteins as previously reported for 1QJP (Figure S5A) [113,114]. Pockets 3 and 6 were predicted to be quite conserved (Figure S5A). Although the amino acid residues of pocket 2 were predicted as unconserved, superimposition of the 1P4T structure with the ROB2 model revealed that pocket 2 shared a similar position/region with another binding cavity of 1P4T. The alignment also revealed that pocket 1 of the WSP shared the same position/region as the “loop site” of the template structure (1P4T).

### 3.4. Selecting Other Anti-Onchocerca sp. Compounds

Compounds that have been experimentally shown to inhibit other *Onchocerca* species were identified. *M. lucida* was shown to possess anthelmintic activities with IC_50_ values ranging from 7.8 to 15.63 μg/mL on *O. ochengi* [115]. The major chemical constituents of *M. lucida* are 1,8-cineole, and α-terpinyl acetate, sabinene and β-pinene [116]. An essential oil from *Cyperus articulatus* L. Additionally exhibited anti-*Onchocerca* activity with IC_50_ values of 23.4 μg/mL, 23.4 μg/mL and 31.25 μg/mL on microfilariae, adult male worms and adult female worms of *Onchocerca ochengi*, respectively [117]. The essential oils of *C. articulatus* are significantly dominated by α-pinene, β-maaliene, β-pinene, γ-patchoulene, cadinol, carveol, caryophyllene, caryophyllene oxide, cedrol, copaene, cyperotundone, germacrone, trans-pinocarveol and piperitone [118,119].

Voacangine and voacamine, isolated from *Voacanga africana* demonstrated strong inhibition against both the microfilariae and adult male worms of *O. ochengi* with IC_50_ values ranging from 2.49 to 9.07 µM. *V. africana* is used traditionally in Cameroon for the treatment of onchocerciasis [120]. Lantadene A (rehmannic acid) isolated from *Lantana camara* leaves also demonstrated strong inhibition of *O. ochengi* with IC_50_ values of 7.85 μg/mL for adult male worms, 10.38 μg/mL for adult female worms and 10.84 μg/mL for microfilariae [121]. 

The anthelmintic effect of ellagic acid from axlewood tree, *Anogeissus leiocarpus*, showed strong activity against *O*. *ochengi* after 48 h, producing LC_50_ values of 0.09 mM and 0.03 mM in adult worms and microfilariae, respectively [122]. Polycarpol and 3-*O*-acetyl aleuritolic acid also demonstrated significant inhibitory activities on the viability of adult male worms of *O. gutturosa* with viability reduction values of 80% and 64.8%, respectively [123]. The structures of the 23 aforementioned anti-*Onchocerca* compounds were obtained from PubChem [124,125,126]. 

### 3.5. Pre-Filtering of Compound Libraries 

A total of 42,883 ligands obtained from three databases were used as the screening library, in addition to **23** compounds obtained from literature as *Onchocerca* species inhibitors. The compounds were divided into three categories: the African natural products (Afro), the traditional Chinese natural products (TCM) and the inhibitors of other *Onchocerca* species comprising **7722**, **35,161** and **23** compounds, respectively. Prior to virtual screening, the Afro and TCM compounds were filtered by eliminating ligands with molecular weights greater than 600 g/mol and less than 150 g/mol. For the Afro dataset, **4440** compounds passed this criterion, with **821** and **3619** from AfroDB and NANPDB, respectively. The TCM library was also filtered by removing duplicates. A total of **25,212** compounds from the TCM dataset passed these criteria.

### 3.6. Molecular Docking Studies

Two stages of virtual screening were employed in this study: the first involved the screening of all the compounds against the ROB2 structure, while the second stage involved the virtual screening of the top **20** shortlisted potential leads each of the two libraries (TCM and Afro) against the ITAS1 and MOD2 structures. The top **11** Oncho related compounds were also screened against models ITAS1 and MOD2 in the second stage.

#### 3.6.1. Virtual Screening against Model ROB2

AutoDock Vina [87] was employed for the virtual screening process. AutoDock Vina requires a pre-calculated grid map to be defined to surround the region of interest composed of the binding site. The pre-filtered library comprising a total of **29,652** compounds and **23** *Onchocerca* spp. inhibitors were successfully screened against the energy minimised WSP. 

A binding affinity threshold of −7.0 kcal/mol has been reported to significantly differentiate between putative specific and nonspecific protein–ligand bonds [127]. Other studies have also used customized thresholds such as the top 10% or even more stringent thresholds [62,128]. The top 10% of the Afro library with high binding affinity to the WSP (**444** compounds) were selected for downstream analysis. The binding affinity of the **444** compounds ranged from −9.8 kcal/mol to −11.3 kcal/mol. Among the Afro library, NANPDB470 demonstrated the highest binding affinity to the WSP with binding energy of −11.6 kcal/mol. Both NANPDB468 and ZINC000095486062 had a binding energy of −11.3 kcal/mol. Additionally, NANPDB2316, NANPDB5642 (Figure S6A), NANPDB386 had a good binding energy of −11.2 kcal/mol, followed by ZINC000095486235 and NANPDB2363, both with an energy of −11.1kcal/mol. A total of **77** Afro compounds (**5** and **72** compounds from AfroDB and NANPDB, respectively) including NANPDB386, NANPDB470, NANPDB468 and NANPDB2316 which had appreciable binding energies were not considered since they did not dock deep into the binding pockets of the WSP. 

**Figure 4:**
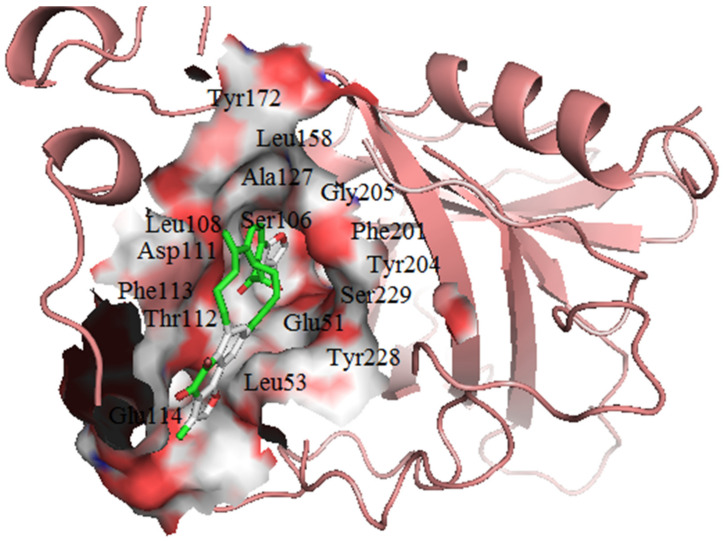
Cartoon representation of the WSP (model ROB2) in complex with ZINC000095913861. The binding sites are represented as surface and the ligands are shown as sticks.

For the TCM library, the top 1% of compounds with high binding affinity to the WSP (**252** compounds) were considered for further analysis. The binding affinities of the selected TCM compounds ranged from −10.3 to −12.7 kcal/mol. A total of **12** compounds did not dock deep into the binding cavities of the WSP and were therefore eliminated. From the molecular docking results, ZINC000095913861 (Figure 4) had the lowest binding energy (−12.7 kcal/mol) with the WSP followed by ZINC000070455413 and ZINC000103584225 with energies of −11.7 and −11.6 kcal/mol, respectively (Table 3). ZINC000085530783 and ZINC000103580868 both had a binding energy of −11.5 kcal/mol. Additionally, both ZINC000085594065 and ZINC000100822646 demonstrated good binding affinity with energy of −11.4 kcal/mol (Table 3).

For the *Onchocerca* inhibitors, compounds with binding energies less than −7.0 were considered. A total of **15** compounds passed this criterion and were considered for downstream analysis. Acetylaleuritolic acid demonstrated the highest binding affinity of −10.3 kcal/mol with the WSP (Table 3). Voacamine, rehmannic acid (lantadene A), polycarpol and ellagic acid also demonstrated good binding affinities with the WSP with energies of −9.7, −9.5, −9.2 and −8.6 kcal/mol, respectively. Eight compounds including copaene, carveol, piperitone, trans pinocarveol, eucalyptol, sabinene, beta pinene and alpha pinene were eliminated since they had binding energies higher than the −7.0 kcal/mol threshold (they had binding energies of −6.6, −6.6, −5.9, −5.8, −5.7, −5.4, −5.3 and −5.2 kcal/mol, respectively).

After structurally visualizing the screening results via PyMOL 1.7.4.5, most of the ligands docked into pockets 1 and 2 (Figure S5C The two binding pockets could be pharmacologically targeted for the design of novel inhibitors, which necessitate the need to investigate if they may play any pivotal role in combination therapy or polypharmacology. Combination therapy of antibiotics successfully depletes *Wolbachia* and reduces treatment duration [129,130,131].

#### 3.6.2. Redocking with Models ITAS1 and MOD2

For the TCM library, all the top **20** compounds had binding energies less than the pre-specified cut-off of −7.0 kcal/mol when docked against both ITAS1 and MOD2 protein structures (Table S8) [127]. For the ITAS1 model, the binding energies ranged from −7.3 to −10.2 kcal/mol with ZINC000095913861 demonstrating the highest affinity to the ITAS1 model (−10.2 kcal/mol) (Table S8). ZINC000095913861, which had the highest binding affinity to the ROB2 and ITAS1 structures, also had the highest affinity to the MOD2 structure with an energy of −11.9 kcal/mol. The binding energies of the top **20** TCM leads with MOD2 ranged from −8.4 to −11.9 kcal/mol (Table S8). 

For the Afro library, the top **20** compounds were also observed to have binding energies lower than the −7.0 kcal/mol threshold [127], ranging from −7.1 to −8.8 and −8.5 to −10.4 kcal/mol for the ITAS1 and MOD2 structures, respectively (Table S8). ZINC000095486235 had binding energies of −9.4 and −7.1 kcal/mol with the MOD2 and ITAS1 models, respectively. NANPDB4566 demonstrated good binding affinities with the MOD2 and ITAS1 structures with energy values of −9.3 and −7.9 kcal/mol, respectively (Table S8). 

For the *Onchocerca* spp related compounds, only three compounds comprising acetylaleuritolic acid, polycarpol and rhemannic acid had binding energies lower than the −7.0 kcal/mol threshold [127] when virtually screened against the ITAS1 and MOD2 structures (Table S8). Acetylaleuritolic acid had binding energies of −9.1 and −7.9 kcal/mol with MOD2 and ITAS1, respectively (Table S8). Polycarpol also demonstrated good binding affinity with ITAS1 and MOD2 with energies of −8.2 and −8.8 kcal/mol, respectively (Table S8). Rhemannic acid also had binding affinities of −8.4 and −8.9 kcal/mol with ITAS1 and MOD2, respectively (Table S8). For the ITAS1 model, cardinol, germacrone and cedrol also passed the threshold with binding affinities of −7.2, −7.1 and −7.1 kcal/mol, respectively. For the MOD2 structure, ellagic acid and voacangine had binding energies of −8.2 and −7.1 kcal/mol, respectively. The binding energies ranged from −6.3 to −8.4 and −5.8 to −9.1 kcal/mol for the top **11** compounds when screened against the ITAS1 and MOD2 models, respectively (Table S8). 

### 3.7. Visualization of Protein–Ligand Interactions

**Figure 5:**
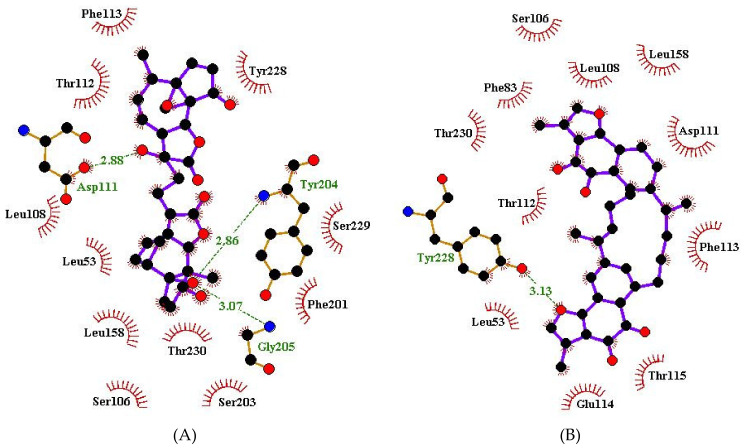
Two-dimensional plots of the WSP-ligand interactions generated using LigPlot+. Interaction profiles of (**A**) WSP-NANPDB5642 complex and (**B**) WSP-ZINC000095913861 complex. Hydrophobic contacts are represented as red spoke arcs, hydrogen bonds are represented as green dash lines and the ligands are colored purple.

The protein–ligand interactions of the WSP were visualized using LigPLOT+ v1.4.5 [92]. Compounds which did not interact with WSP via any hydrogen bonds were not considered for further analysis. For the TCM library, a total of **33** compounds did not form any hydrogen bonds with the WSP and were thus not considered for downstream analysis. ZINC000095913861 which had the highest binding affinity (−12.7 kcal/mol) and formed a hydrogen bond with Tyr228 of length 3.13 Å and hydrophobic contacts with Leu53, Phe83, Ser106, Leu108, Asp111, Phe113, Glu114, Thr115, Leu158 and Thr230 (Figure 4 and Figure 5B and Table 3). ZINC000070455413 interacted with Gly91 (2.81 Å), Tyr43 (3.06 Å) and Arg45 (3.23 Å) via hydrogen bonds and Cys42, Phe90, Arg98, Tyr135, Val139, Pro147, Tyr148, Ser185, Asp187, Phe195 and Met238 via hydrophobic bonds. ZINC000103584225 formed 3 hydrogen bonds with Glu114, Glu171 and Ala206 of bond lengths 3.04, 3.21 and 2.9 Å, respectively. It also formed hydrophobic contacts with Ser106, Leu108, Asp111, Thr112, Phe113, Ile121, Ala127, Leu158, Tyr172, Ser203, Tyr204 and Gly205. ZINC000085530783 interacted via hydrogen bonding with Arg98 (2.94 Å) and Lys193 (2.97 Å); and formed hydrophobic bonds with Arg45, Asp100, Tyr135, Tyr136, Asp137, Val138, Val139, Ile146, Pro147, Tyr148, Ser185, Asp187 and Phe195. It is worth noting that ZINC000085532042, NANPDB512 and NANPDB769 formed 12, 10 and 9 hydrogen bonds, respectively, with the WSP. The high numbers of hydrogen bonds and relatively short bond distances indicate strong binding and could make these ligands interesting compounds to further investigate [132].

For the Afro library, a total of **47** compounds did not form any hydrogen bond interaction with the WSP. NANPDB5642, which had binding energy of −11.2 kcal/mol, formed 3 hydrogen bonds with Tyr204, Gly205 and Asp111, with bond lengths of 2.86, 3.07 and 2.88 Å, respectively (Figure 5A and Figure S6A, Table 3). Hydrophobic interactions with Leu53, Ser106, Leu108, Thr112, Phe113, Leu158, Phe201, Ser203, Tyr228, Ser229 and Thr230 were observed (Figure 5A and Table 3). NANPDB2874 docked into pocket 1, interacted via hydrogen bonds with residues Arg45 (3.02 and 3.33 Å), Gly91 (3.25 Å), Tyr135 (2.81 Å) and Lys181 (3.26 Å), and made hydrophobic contacts with Asp100, Tyr136, Val139, Pro147, Tyr148, Ser185 and Phe195. 

ZINC000095486235 which had a binding energy of −11.1 kcal/mol, and was observed to interact with Gly91 via hydrogen bonding with a length of 2.98 Å and Cys42, Arg45, Arg98, Asp100, Tyr135, Tyr148, Ser185, Lys193, Phe195 and Met238 via hydrophobic contacts (Table 3, Figures S6B and S7A). NANPDB513 formed four hydrogen bonds with the WSP by interacting with Glu114, Thr115, Ala116 and Tyr204 of bond lengths 2.88, 3.23, 3.24 and 3.07 Å, respectively. NANPDB4566 also interacted with two WSP residues comprising Tyr135 and Lys181 with hydrogen bond lengths of 2.9 and 2.8 Å, respectively. It also formed hydrophobic bonds with Arg45, Arg98, Asp100, Tyr136, Asp137, Val138, Val139, Ile146, Pro147, Tyr148, Ser185, Phe195 and Glu234. ZINC000035941652 interacted with the WSP via four hydrogen bonds with residues Tyr135 (3.03 Å), Lys181 (3.17 Å), Ser185 (3.09 Å) and Glu234 (3.28 Å). 

For the *Onchocerca* species inhibitors, the compounds comprising beta maaliene, caryophyllene and gamma putchoulene did not form any hydrogen bonds with the WSP and were thus not considered for further analysis. Acetylaleuritolic acid formed 7 hydrogen bonds with Glu114 (3.18 Å), Thr115 (2 hydrogen bonds, both with a length of 2.93 Å), Ala116 (3.18 Å), Tyr204 (2.81 Å), Gly205 (3.1 Å) and Ser229 (2.8 Å) [Table 3, Figures S6C and S7B]. It also formed hydrophobic contacts with Phe83, Ser106, Leu108, Asp111, Thr112, Phe113, Glu114, Thr115, Ala116, Ser203, Tyr204, Gly205, Tyr228, Ser229 and Thr230 (Table 3 and Figure S7B). Lantadene A (rehmannic acid) interacted with the WSP via one hydrogen bond with Ser106 (3.09 Å). Hydrophobic interactions with Ser106, Leu108, Asp111, Thr112, Phe113, Glu114, Ala116, Ala127, Leu158, Phe201, Tyr204, Tyr228, Ser229 and Thr230 were observed. Polycarpol also interacted with the WSP via 2 hydrogen bonds with Ser106 and Thr112 with bond lengths of 2.7 and 3.0 Å, respectively. It formed hydrophobic contacts with Leu53, Ser106, Thr112, Phe113, Ala116, Ala127, Phe201, Ser203, Tyr204, Tyr228, Ser229 and Thr230.

In addition, Arg45, Tyr135, Tyr148, Ser185 and Phe195 were involved in majority of the active site interactions in pocket 1 and therefore may possibly be key residues required for stronger interactions and stability of the ligands.

### 3.8. ADMET Prediction

#### 3.8.1. ADME Prediction of Lead Compounds

SwissADME was employed to determine the physicochemical and pharmacokinetic properties of the shortlisted compounds [91]. Lipinski’s and Veber’s rules were applied to filter the compounds. Lipinski’s rule requires a drug-like molecule not to have more than 10 H-bond acceptors, not more than 5 H-bond donors, a molecular mass less than 500 g/mol and an octanol-water partition coefficient (log P) less than five. Veber’s rule however, requires a drug-like molecule to have a topological polar surface area (TPSA) less than or equal to 140 Å^2^ and less than 11 rotatable bonds [133]. 

Although natural products are known to exhibit different physiochemical characteristics that do not adhere to Lipinski’s and other ADMET rules, a study has also reported that over 50% of natural products had desirable drug-like properties upon predicting the pharmacokinetic properties and drug-likeness of these natural products [134]. A study, which compared **1040** natural products with known anti-malaria compounds revealed that there was no significant difference between the number of rotatable bonds of the natural products and the known anti-malaria compounds (mostly synthetic compounds) [134]. About 70% of the natural products also fell below the upper limit for the TPSA (140 Å^2^). In other words, most of the safe natural products obey the Veber’s rule [134]. Although most natural products do not obey the molecular weight and hydrogen bond acceptor and/or donor components of the Lipinski’s rule, they were largely found to obey the logP component of the rule [134,135]. Since natural products are amenable to further improvements, this study demonstrates the feasibility of obtaining natural products with the required physicochemical properties for drug discovery. For the Afro library, **164** compounds failed Lipinski’s and/or Veber’s rules. A total of **160** failed only Veber’s rule while **118** failed only Lipinski’s rule. 

NANPDB2874 with TPSA of 109.05 Å^2^ was predicted as moderately soluble with high gastrointestinal absorption (GI) (Table S6). NANPDB5642 failed the Veber’s rule due to its high TPSA (147.43 Å^2^), was predicted as a Pgp substrate, a non-BBB permeant and did not inhibit any CYP enzyme. The blood–brain barrier (BBB) is a highly selective semipermeable membrane that prevents solutes in the blood from non-selectively crossing into the extracellular fluid of the central nervous system which houses neurons. *O. volvulus* microfilariae have not been found in the brain parenchyma [7], however onchocerciasis-associated epilepsy cases may require BBB permeation [8,136,137]. Multidrug-resistance proteins have been shown to limit access of antiepileptic drugs into brain parenchyma leading to seizures which are refractory to treatment [136]. NANPDB4566 with TPSA value of 59.67 Å^2^ was predicted as moderately soluble with high GI absorption and as CYP2C19, CYP2C9, CYP2D6 and CYP3A4 inhibitors. NANPDB4566 was also predicted as a BBB permeant and a Pgp substrate (Table S6). 

For the TCM library, **73** compounds failed the ADMET testing. A total of **44** compounds including ZINC000070455413, ZINC000085530783 and ZINC000028642721 violated two or more conditions of the Lipinski’s rule while **57** compounds violated Veber’s rule. A total of **28** compounds including ZINC000070455413, ZINC000070455574, ZINC000095914553, ZINC000003984030 and ZINC000085593983 violated Lipinski’s and Veber’s rules simultaneously. ZINC000095913861 had a TPSA of 94.56 Å^2^, was predicted as a non-BBB permeant, non-Pgp substrate and did not inhibit any of the determined CYP isoenzymes. However, it was predicted to have a low GI absorption and to be poorly soluble (Table S6). ZINC000085594065 and ZINC000100822646, with TPSA values of 137.41 and 135.29 Å^2^, respectively were also predicted as poorly soluble with low GI absorption (Table S6). ZINC000085594065 was predicted as CYP1A2 and CYP2C9 inhibitors while ZINC000100822646 only inhibited CYP2C9. They were both predicted as non-BBB permeants and non-Pgp substrates. ZINC000103543220 with TPSA of 107.35 Å^2^ was predicted as a Pgp substrate and as CYP1A2 and CYP2C9 inhibitors.

#### 3.8.2. Toxicity Profiles of Lead Compounds

The toxicity profiles of the selected hits were determined using OSIRIS DataWarrior 5.0.0 (Table S7) [84]. ZINC000070455413, ZINC000085530783, ZINC000100822646, NANPDB5642, ZINC000095486235, NANPDB2874, ZINC000035941652, NANPDB4566, NANPDB513 and acetylaleuritolic acid were predicted as non-mutagenic, non-tumorigenic, non-irritant and have no reproductive effects (Table S7). ZINC000095913861 was predicted as non-mutagenic, non-tumorigenic and non-irritant. However, it was predicted to have high reproductive effect (Table S7). ZINC000103584225 was also predicted to be highly mutagenic and as non-tumorigenic, non-irritant and with no reproductive effect. Rhemannic acid and Polycarpol were predicted as highly irritant with no reproductive effect. They were both also predicted as non-mutagenic and non-tumorigenic (Table S7). Finally, ZINC000085594065 was predicted to have low mutagenicity, low tumorigenicity and high irritancy. It was also predicted to have high reproductive effect (Table S7). 

### 3.9. Prediction of Biological Activity of Compounds

The potential biological activities of the identified biomolecules were predicted using the Prediction of Activity Spectra of Substances (PASS) [54,55,56]. Most of the compounds were predicted as antibacterial, antiparasitic and antihelmintic. ZINC000095913861 with binding affinity of −12.7 kcal/mol was predicted as antiparasitic (Pa: 0.464 and Pi: 0.020) and antibacterial (Pa: 0.234 and Pi: 0.093). ZINC000100822646 was predicted as an anthelmintic (anti-nematode) with Pa and Pi values of 0.522 and 0.013, respectively. It was also predicted to possess antiparasitic, antihelmintic, antirickettsial and antibacterial activity. NANPDB2874 (naamidine A) with binding energy of −11.1 kcal/mol was predicted as a caspase 3 stimulant with Pa and Pi of 0.873 and 0.004, respectively. It was also predicted as an apoptosis agent (Pa: 0.515 and Pi: 0.038) and an interleukin 4 antagonist (Pa: 0.152 and Pi: 0.047).

ZINC000095486235, also with binding energy of −11.1 kcal/mol, was predicted as caspases 3 and 8 stimulants and as an apoptosis agent. NANPDB4566 (coladonin) was predicted as a caspase 3 stimulant (Pa: 0.868 and Pi: 0.004), apoptosis agonist (Pa: 0.736 and Pi: 0.012), immunosuppressant (Pa: 0.704 and Pi: 0.016), dermatologic (Pa: 0.673 and Pi: 0.009), antipruritic (Pa: 0.672 and Pi: 0.010) and caspase 8 stimulant (Pa: 0.654 and Pi: 0.004). It was also predicted as antibacterial, antiparasitic and antihelmintic (anti-nematode). WSP has been shown to possess anti-apoptotic activity, however, reduced caspase 3 activity has also been shown to inhibit apoptosis in human neutrophils [128,129]. Caspase 8 is required for caspase 3 activation, thus, the activation or stimulation of caspases 3 and 8 could promote apoptosis [138,139]. 

A total of **5** compounds including NANPDB2874, NANPDB2877, NANPDB4599, NANPDB2876 and NANPDB3435 were predicted as interleukin 4 (IL-4) antagonists. Interleukin 4 has been reported to play a very crucial role in the protective immune response to *O. volvulus* and the absence of IL-4 has been shown to prevent the development of a corneal disease [140,141]. *Wolbachia* from *O. volvulus* possesses molecules with lipopolysaccharide-like activities that signal immune response through toll-like receptor 4 (TLR4) [142]. Other compounds including ZINC000100822646, ZINC000042876996, ZINC000095910593, ZINC000095911361, NANPDB1649, NANPDB4605, NANPDB4312 and ZINC000095486126 were predicted as anti-rickettsials which can lead to *Wolbachia* depletion since the bacterium belongs to the genus *Rickettsia*.

ZINC000100822646 (palmidin C) has been identified in *Cassia fistula Linn* (Senna), an ayurvedic Indian medicine [143]. The pulp of the pod and the leaves of Senna are used for treating helminth infections [144]. The anthelmintic activity of fruit pulp, stem bark and seeds of Senna have been studied in previous works and the extracts were found to paralyze and kill *Pheretima posthuma* [145,146]. Hydro-alcohol extracts of leaves of *C. fistula Linn* demonstrated remarkable inhibition of the bacterial growth of *Staphylococcus aureus* [147], and *Wolbachia* has been reported to share similarity with *Staphylococcus aureus* infection (in mice) in the initiation of TLR2/6, phagocytosis-mediated induction of NETosis [148]. Additionally, aqueous extract of *Senna occidentalis* has been shown to completely inhibit the hatching of eggs and caused 96.3% inhibition of larval development of *Haemonchus contortus* [144]. It is reported that Ivermectin selection changes the frequency of beta-tubulin alleles in both *H. contortus* and *O. volvulus* [149]. 

ZINC000042876996 (withanone) is an active constituent of Ashwagandha (*Withania somnifera*), which has been shown to possess immunoprophylactic properties against *Brugia malayi* in *Mastomys coucha* (mice) [150,151,152]. ZINC000040421861 (isovouacapenol D), a derivative of isovouacapenol, which is found in *Caesalpinia pulcherrima* [153,154,155] and has been found in previous studies to possess anthelmintic activity. Additionally, *Carex baccans* has shown strong in vivo anthelmintic activity against *Hymenolepis diminuta* [156]. ZINC000032052445 (pallidol) is a major constituent of the seeds and leaves of *Carex* species [157,158]. 

ZINC000001531664 (ginkgetin) and ZINC000003197535 (isoginkgetin) originally isolated from *Ginkgo biloba* (Ginkgoaceae) possess inhibitory effects on nuclear factor-kappa B (NF-κB) activation. Ginkgetin demonstrated inhibition with an IC_50_ value of 7.5 μM [159]. ZINC000003984030 (amentoflavone) extracted from *Selaginella tamariscina* also inhibits NF-kappaB activation in macrophages [160]. *Wolbachia* induces TLR2 activation in resident macrophages in the corneal stroma which in turn induces NF-κB cells translocation to the nucleus and the production of proinflammatory and chemotactic cytokine [27,161]. Ginkgetin, isoginkgetin and ochnaflavone (ZINC000028462577) at 10 μM demonstrated suppressive activity against lymphocyte proliferation induced by concanavaline A (Con A) or lipopolysaccharide (LPS) and were found to be irreversible inhibitors of lymphocyte proliferation [162]. Although, ZINC000001531664, ZINC000003197535 and ZINC000028462577 were below the threshold of ADMET properties, their antiwolbachial potential can be investigated.

Similarity search performed via DrugBank revealed that ZINC000035941652 is similar to naringenin (DB03467) and hesperetin (DB01094) with similarity scores of 0.906 and 0.861, respectively. Naringenin and hesperetin have been identified to possess in vitro antifilarial activity on *B. malayi* [163]. Naringenin and hesperetin have shown effective microfilaricidal activity and were observed to inhibit the reduction of MTT by 60% at concentrations of 7.8 μg/mL and 31.2 μg/mL, respectively [163]. Naringenin also demonstrated in vitro macrofilaricidal activity at 125 μg/mL and inhibited female worm motility with an IC_50_ of 2.5 μg/mL [163]. However, when naringenin was tested in vivo at 50 mg/kg, it eliminated 73% and only 31% of the *B. malayi* macrofilariae in the *Meriones unguiculatus* (implanted adult worms) and *Mastomys coucha* (natural infections) models, respectively [163]. When the dose was doubled, naringenin killed 51% of the microfilariae in the *M. coucha* models [163].

### 3.10. Molecular Dynamics Simulation

**Figure 6:**
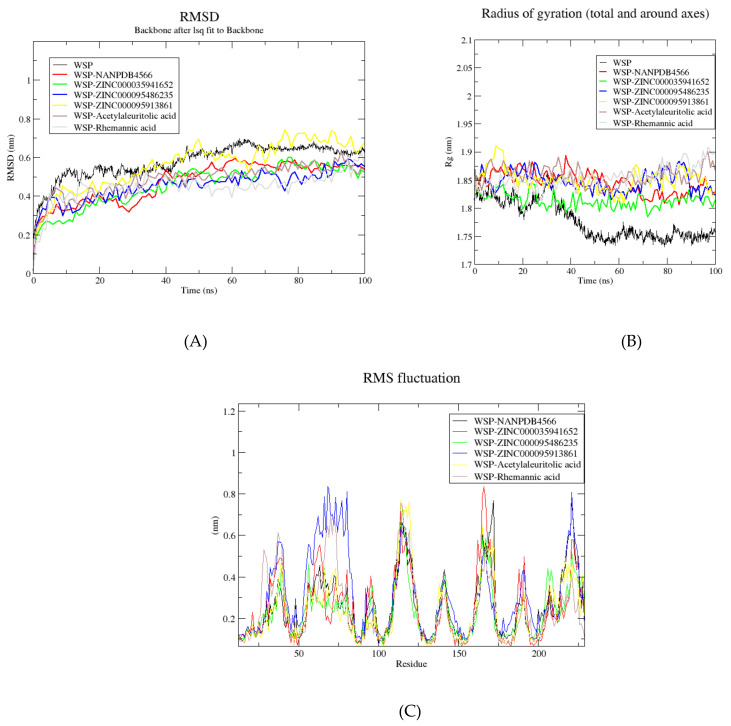
Root mean square deviation (RMSD), root mean square fluctuations (RMSF) and the radius of gyration (Rg) plots of 100 ns molecular dynamics (MD) simulations of the WSP-ligand complexes using GROMACS. (**A**) RMSD graph of the WSP-ligand complexes, (**B**) the Rg versus a time graph of the WSP-ligand complexes and (**C**) analysis of RMSF trajectories of residues of the WSP-ligand complexes. Legends with color codes are provided in each plot.

Based on the analysis performed, six compounds were subjected to molecular dynamics (MD) simulations to further analyze the molecular mechanisms of the protein–ligand interactions. For the *Onchocerca* related library, 2 compounds comprising acetylaleuritolic acid and rhemannic acid were selected. NANPDB4566, ZINC000095486235 and ZINC000035941652 were selected from the Afro library. ZINC000095913861 with the highest binding affinity to the WSP (−12.7 kcal/mol) was selected from the TCM library. The unbound WSP protein was also subjected to MD simulations. The root mean square deviation (RMSD), the radius of gyration (*R_g_*) and the root mean square fluctuation were analyzed for the unbound protein and all the six WSP-ligand complexes (Figure 6A–C). 

To investigate the stability of the WSP-ligand complexes, the RMSD plots of the unbound protein and the complexes were generated (Figure 6A). RMSD is mostly used to determine the differences between the structures during the simulation period and their reference structure [164]. The RMSD trajectory shows the deviation of a protein structure from its reference structure with respect to time [164]. The RMSD plots of the unbound protein and the six WSP-ligand structures experienced similar trends. The RMSDs of all the seven structures rose from 0 to about 10 ns, where stability was observed. For the unbound protein, the RMSD remained stable maintaining an average of about 0.55 nm till about 42 ns where a slight rise to an average of 0.65 nm was observed until the end of the simulation period (Figure 6A). The most fluctuations in RMSD were observed for the WSP-ZINC000095913861 complex, which rose to an average of 0.7 nm from 0 to 50 ns, then fell to an average of 0.6 nm till 70 ns (Figure 6A). A rise in RMSD was observed with an average of 0.68 nm until the end of the 100 ns period (Figure 6A). Rhemannic acid, acetylaleuritolic acid, NANPDB4566, ZINC000095486235 and ZINC000035941652 demonstrated similar RMSD trends and were very stable throughout the 100 ns simulation period (Figure 6A). 

The reasonably invariant *R_g_* plots of the 6 WSP-ligand complexes remained very stable in their compact forms over the course of 100 ns as compared to the unbound WSP (Figure 6B). The *R_g_* values of the unbound WSP showed stability from 0 to about 35 ns with an average of 1.82 nm and then fell until about 50 ns where it remained steady until the end of the 100 ns with an average value of 1.75 nm (Figure 6B). The radius of gyration of a protein is a measure of its compactness. If a protein is stably folded, it will likely maintain a relatively steady value of *R_g_*. If a protein unfolds, its *R_g_* will change over time. The *R_g_* of the WSP-acetylaleuritolic acid complex remained stable throughout the 100 ns simulation period with an average of 1.85 nm (Figure 6B). The WSP-ZINC000035941652 complex demonstrated the lowest and most stable *R_g_* among the protein–ligand complexes, maintaining an average of 1.81 nm (Figure 6B). For the WSP-rhemannic acid complex, the *R_g_* maintained an average value of 1.85 till about 70 ns, where a rise to about 1.875 nm was observed (Figure 6B). Similar *R_g_* plots were observed for ZINC000095486235 and ZINC000095913861, both maintaining an average *R_g_* of about 1.86 nm (Figure 6B).

The RMSF trajectories of the 6 WSP-ligand complexes were also evaluated to investigate the WSP residues contributing to the structural fluctuations (Figure 6C). RMSF is used to analyze the flexibility of different protein regions, and is related to crystallographic B-factors [164]. Higher RMSF values imply greater fluctuations and these protein regions are involved in ligand binding and catalysis [165]. All the 6 WSP-ligand complexes demonstrated some degree of fluctuations at the same regions (Figure 6C). Major fluctuations above 0.4 nm were observed at WSP residue index 27–50, 60–87, 110–130, 160–175, 183–197 and 218–236, implying they could be involved in ligand binding [165] [Figure 6C]. At residue index 60–87, ZINC000095913861 which had the highest binding affinity (−12.7 kcal/mol), caused the highest fluctuation of the WSP with a highest peak of 0.85 nm, followed by rhemannic acid which had its highest RMSF peak at about 0.7 nm (Figure 6C). Similarly, ZINC000095913861 caused the highest fluctuation at residue index 218–236 with the highest peak of 0.85 nm (Figure 6C). The hydrogen bond between ZINC000095913861 and Tyr228, and hydrophobic interaction with Thr230 could account for the high fluctuation recorded at residue index 218–236. Additionally, its interaction with Leu53 and Phe83 could be the cause of the fluctuation observed at index 60–87. At residue index 110–130, all the 6 WSP-ligand complexes exhibited similar peaks with a high RMSF of about 0.75 nm (Figure 6C). For residue index 160–175, ZINC000035941652 demonstrated the highest RMSF at about 0.85 nm, followed by NANPDB4566 with a peak of 0.75 nm. Minor structural fluctuations were also observed at residue index 90–100, 135–150 and 210–220 (Figure 6C).

### 3.11. Molecular Mechanics Poisson-Boltzmann Surface Area (MMPBSA) Computation

#### 3.11.1. Energies Involved in WSP-Ligand Binding

Molecular Mechanics Poisson-Boltzmann Surface Area (MMPBSA) computes the binding free energies of the WSP-ligand complexes. Simulation-based methods such as MMPBSA provide more accurate estimates of protein–ligand binding affinities than other computational approaches such as docking [166]. Investigating the binding free energy of protein–ligand complexes can reveal the ligands with higher binding affinities to the target. Thus, binding free energy computations reveal the binding modes and affinities of the hits in drug design [167]. ZINC000095913861 had the least binding free energy of −111.73 kJ/mol (Table 4). It also demonstrated the least binding energy in the molecular docking study with a value of −12.7 kcal/mol (Table 4). ZINC000095486235 demonstrated the highest binding free energy of −48.899 kJ/mol (Table 4). Acetylaleuritolic acid, NANPDB4566, rhemannic acid and ZINC000035941652 also had binding free energies of −92.468, −91.683, −83.884 and −82.477 kJ/mol, respectively (Table 4). 

Earlier studies suggest that electrostatic and van der Waal’s forces contribute predominantly to the binding energy [94,168]. All the complexes demonstrated very low van der Waal’s energies ranging from −131.255 kJ/mol to −216.596 kJ/mol (Table 4). ZINC000095913861 had the lowest van der Waal energy of −216.596 kJ/mol while ZINC000095486235 demonstrated the highest with van der Waal energy of −131.255 kJ/mol (Table 4). NANPDB4566, rhemannic acid, ZINC000035941652 and acetylaleuritolic acid complexes also demonstrated van der Waal energies of −158.603, −152.808, −148.894 and −137.347 kJ/mol, respectively (Table 4). 

For the electrostatic energy, NANPDB4566 demonstrated the least with −45.067 kJ/mol, followed by ZINC000035941652 (−43.213 kJ/mol), while acetylaleuritolic acid had the highest electrostatic energy of −2.452 kJ/mol (Table 4). Rhemannic acid, ZINC000095913861 and ZINC000095486235 also had electrostatic energies of −25.762, −12.103 and −3.563 kJ/mol, respectively (Table 4). The two-dimensional (2D) structures of the top six compounds are provided in Table 5.

#### 3.11.2. Per-Residue Energy Decomposition

**Figure 7:**
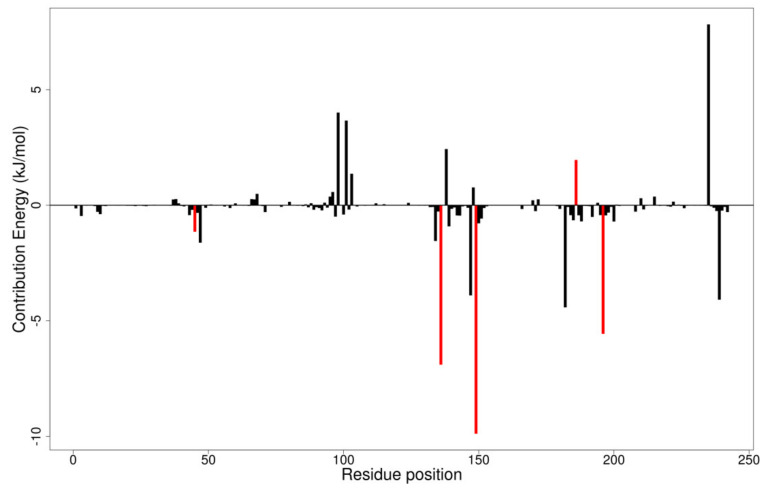
Molecular mechanics/Poisson-Boltzmann surface area (MMPBSA) plot showing the binding free energy contribution per residue of the WSP-NANPDB4566 complex.

MMPBSA method can also be used to investigate the energy contribution of each residue. This involves the decomposition of each residue by including the interactions in which each residue is involved. Residues which contribute energies greater than 5 kJ/mol or less than −5 kJ/mol are worth considering as critical residues for protein–ligand binding [169]. The per-residue energy decomposition computation for each protein–ligand complex was performed (Figure 7 and Figure S8A–E).

From the protein–ligand interaction studies, Arg45, Tyr135, Tyr148, Ser185 and Phe195 were identified as critical for ligand binding in pocket 1. For the WSP-NANPDB4566 complex, Tyr135, Tyr148 and Phe195 were observed to contribute energies less than −5 kJ/mol, which are favorable for ligand binding (Figure 7). Tyr135, Tyr148 and Phe195 contributed individual energies of −6.8971, −9.8840 and −5.5633 kJ/mol, respectively (Figure 7). Glu234 contributed energy of 7.8227, which is considered unfavorable for ligand binding. Arg45 and Ser185 contributed energies of −1.1463 and 1.9588 kJ/mol, respectively (Figure 7). The WSP-ZINC000095486235 complex had favorable energies of −7.8888, −1.8052, −5.7684, −4.1513 and −2.4076 kJ/mol for Tyr43, Tyr135, Ile146, Tyr148 and Phe195, respectively. Arg45, Asp100 and Ser185 contributed positive energy values of 12.1903, 6.1679 and 1.7945, respectively (Figure S8A). For the WSP-ZINC000035941652 complex, Arg45, Tyr135, Tyr148 and Phe195 contributed energies of −4.0926, −5.5091, −9.5055 and −4.0762 kJ/mol, respectively (Figure S8B). Arg98 (10.5112 kJ/mol), Asp100 (5.2732 kJ/mol), Asp137 (5.1856 kJ/mol) and Ser185 (0.4530 kJ/mol) contributed energies which were unfavorable for ligand binding (Figure S8B). Arg45, Tyr135, Tyr148 and Phe195 are worthy of further studies to elucidate their role in ligand binding in pocket 1.

For the WSP-ZINC000095913861 complex, Glu51, Phe113, Arg199 and Phe201 contributed individual energies of 6.7173, −8.5824, 6.2146 and −5.6586, respectively (Figure S8C). For the WSP-acetylaleuritolic acid, Leu53 (−5.8137 kJ/mol), Leu108 (−5.9892 kJ/mol), Tyr228 (−7.1338kJ/mol) contributed favorably to ligand binding, while Glu51 contributed a positive energy of 5.3811 kJ/mol (Figure S8D). Phe83 and Arg199 contributed favorably to rhemannic acid’s binding to the WSP with energies of −5.7488 and −6.2429 kJ/mol, respectively (Figure S8E). However, Glu51 was observed to contribute a very high positive energy of 18.2885 kJ/mol which is considered unfavorable to the binding of rhemannic acid (Figure S8E). From the per residue energy decompositions, Glu51 is likely to be very critical to ligand binding in pocket 2.

### 3.12. Experimental Evaluation of the Anti-Onchocercal and Antibacterial Activity of Lantedene A, Polycarpol and 3-Acetylaleuritolic Acid

The compounds 3-*O*-acetylaleuritolic acid, polycarpol and lantedene A (rhemannic acid) have been experimentally shown to possess antifilarial activity [121,123]. Lantedene A, a methylene chloride extract of *Lantana camara* (a Cameroonian medicinal plant used to treat onchocerciasis) was tested in vitro on bovine model parasite, *O. ochengi* as well as *L. loa* microfilariae [121]. Lantedene A inhibited *O. ochengi* adult males and adult females with IC_50_ values of 7.85 and 10.38 μg/mL, respectively, and was suggested for the treatment of onchocerciasis [121]. It also inhibited *L. loa* microfilariae at an IC_50_ of 20.13 μg/mL [121].

A quantity of 12.5 μM of polycarpol and 3-*O*-acetyl aleuritolic acid extracted from *Polyalthia suaveolens* (Annonaceae) and *Discoglypremna caloneura* (Euphorbiaceae), respectively were tested on adult male worms of *O. gutturosa* in triplicates using Amocarzine as a positive control compound [123]. Formazans’ accumulation inhibition assay was also performed to test the worm viability activity of the compounds. Polycarpol and 3-*O*-acetylaleuritolic acid caused 80.0 and 64.8% of inhibition of the accumulation of formazan, whereas the positive control (Amocrazine) inhibited only 48.6% of MTT reduction [123]. 3-*O*-acetylaleuritolic acid isolated from *Spirostachys africana* was tested on various bacteria species for antibacterial activity using micro-dilution method [170]. The 3-*O*-acetylaleuritolic acid demonstrated strong bactericidal activity on *Staphylococcus aureus*, *Salmonella typhi*, *Vibrio cholera*, *Escherichia coli*, *Shigella dysentery*, *Shigella flexneri* and *Shigella boydii*, with minimum inhibition concentration (MIC) of 50, 50, 50, 50, 50, 100 and 100 μg/mL, respectively [170]. Furthermore, 3-*O*-acetylaleuritolic acid was not cytotoxic to Vero cell lines (African green monkey) in vitro at IC_50_s greater than 400 μg/mL [170].

## 4. Implications and Future Outlook

Since the structure of the *Wolbachia* surface protein (WSP) of *O. volvulus* has not been experimentally elucidated, this study generated a reasonably good 3D structure of WSP by employing homology modelling techniques using Modeller, I-TASSER and Robetta. To the best of the authors’ knowledge, only four structures of the *Wolbachia* surface protein in *Drosophila melanogaster*, *Asobara tabida*, *B. malayi* and *Exorista sorbillans* have been modelled (Table S9) [26,171,172]. This study is the first attempt to model the WSP of *O. volvulus* structure and to virtually screen small molecules against the WSP. The predicted structure provides an avenue to elucidate *Wolbachia*-host interactions, promote mutation studies and catalytic site analysis. Even though, natural products have been shown as potent therapeutic molecules, the use of natural product databases to aid in unravelling novel antiwolbachial molecules is underexploited. This study complements current efforts dedicated to the identification of *O. volvulus* inhibitors by targeting *Wolbachia*. These molecules are indispensable in the race against finding a cure as well as eradication of onchocerciasis. A comprehensive pharmacoinformatic approach aimed at identifying novel *O. volvulus* inhibitors targeting the *Wolbachia* surface protein (WSP) was reported in this study. The study carefully predicted drug-like compounds, which was reinforced with machine-learning based anthelmintic and antibacterial activity predictions. MD simulations, along with MMPBSA computations were also employed in this study. The study also suggests the pharmacological targeting of the two identified binding pockets to ascertain if they play any major role in combination therapy. The predicted potential inhibitors could be used as fragments for de novo design of druglike filaricides. Additionally, these compounds are recommended to be tested both in vitro and in vivo to ascertain their anthelmintic activity for further characterization as potent future inhibitory molecules. The study also highlights the potential of repurposing existing compounds as potential inhibitory molecules of *O. volvulus* to aid in the search for therapeutic biomolecules.

## 5. Conclusions

WSP is the major protein in the *Wolbachia* endobacterium and a target for filarial infection and disease control. Lack of experimentally determined structure of WSP, necessitated the modelling of a reasonably validated protein structure. Molecular informatics approaches including molecular docking, physicochemical and pharmacological profiling were employed in the identification of six potential inhibitory small molecules against WSP comprising acetylaleuritolic acid, rhemannic acid, ZINC000095913861, NANPDB4566 (coladonin), ZINC000095486235 and ZINC000035941652. MD simulations and MMPBSA computations revealed new insights into the binding mechanisms including novel residues critical for binding. The natural product molecules were predicted as antibacterial, antiparasitic, anthelmintic and anti-rickettsials. They were also predicted to have good pharmacokinetic properties, negligible toxicity and druglike. Therefore, the potential exists for these molecules to be tested experimentally to ascertain their antiwolbachial activity. The scaffolds identified can be optimized to serve as initial templates for the design of novel filaricides.

## Figures and Tables

**Table 1:** Ramachandran plot statistics for the three best models from each modelling technique. For all the three models, the number of end residues (excluding Gly and Pro) = two, Glycine residues = 22, Proline residues = 11 and the total number of residues = 241.

MODEL	MOD2		ITAS1		ROB2	
	No. of Residues	Percentage	No. of Residues	Percentage	No. of Residues	Percentage
Most favored regions [A, B, L]	170	82.5	111	53.9	169	82.0
Additionally allowed regions [a, b, l, p]	34	16.5	83	40.3	32	15.5
Generously allowed regions [~a, ~b, ~l, ~p]	2	1.0	8	3.9	4	1.9
Disallowed regions	0	0.0	4	1.9	1	0.5
Non-glycine and non-proline residues	206	100.0	206	100.0	206	100.0

**Table 2:** Top 6 binding pockets of ROB2 predicted by CASTp and the amino acid residues lining the pockets. Solvent accessible (SA) values are shown. The volumes and areas of the binding site cavities are measured in Å^3^ and Å^2^, respectively.

POCKET	VOLUME (SA)/Å^3^	AREA (SA)/Å^2^	RESIDUES
1	1013.6	637.3	Ala36, Glu38, Thr40, Ser41, Cys42, Tyr43, Ile44, Arg45, Gln47, Ala89, Phe90, Gly91, Tyr92, Arg98, Val99, Asp100, Tyr135, Tyr136, Val138, Val139, Ile146, Pro147, Tyr148, Val149, Lys181, Ser185, Asp187, Lys193, Leu194, Phe195, Glu234, Met238
2	954.6	672.9	Glu51, Leu53, Asp77, Leu78, Tyr79, Lys80, Ala81, Phe83, Ala85, Leu104, Tyr105, Ser106, Leu108, Asp111, Thr112, Phe113, Glu114, Leu125, Ala127, Leu128, Ser129, Ala156, Leu158, Tyr172, Arg199, Phe201, Ser203, Tyr204, Gly205, Ala206, Tyr228, Ser229, Thr230
3	138.6	86.3	Ile140, Glu141, Asp142, Met143, Pro144, Ile146
4	103.1	58.6	Tyr157, Leu158, Ser159, Lys170, Glu171, Tyr172, Gly173, Phe174
5	270.8	282.2	Phe52, Pro54, Phe55, Glu58, Ile59, Gly64, Ala65, Lys66, Lys67, Asn73, Val74, Val231
6	164.5	144.8	Thr15, Val18, Thr19, Ile35, Phe90, Tyr92, Met94, Val99, Ile101, Val132

**Table 3:** Binding energies and interactions of some selected compounds which docked deep into the WSP.

Compound	Source	Binding Energy (kcal/mol)	Hydrogen Bonds [H-Bond Length (Å)]	Hydrophobic Contacts
ZINC000095913861	TCM	−12.7	Tyr228 (3.13)	Leu53, Phe83, Ser106, Leu108, Asp111, Thr112, Phe113, Glu114, Thr115, Leu158, Thr230.
ZINC000070455413	TCM	−11.7	Tyr43 (3.06), Arg45 (3.23), Gly91 (2.81).	Cys42, Phe90, Arg98, Val139, Tyr135, Pro147, Tyr148, Ser185, Asp187, Phe195, Met238.
ZINC000103584225	TCM	−11.6	Glu114 (3.04), Glu171 (3.21), Ala206 (2.9).	Ser106, Leu108, Asp111, Thr112, Phe113, Ile121, Ala127, Leu158, Tyr172, Ser203, Tyr204, Gly205.
ZINC000085530783	TCM	−11.5	Arg98 (2.94), Lys193 (2.97).	Arg45, Asp100, Tyr135, Tyr136, Asp137, Val138, Val139, Ile146, Pro147, Tyr148, Ser185, Asp187, Phe195.
ZINC000085594065	TCM	−11.4	Thr40 (2.91), Arg45 (2.94), Ser185 (2.57; 3.18).	Glu38, Ser41, Cys42, Tyr43, Gly91, Arg98, Tyr135, Tyr148, Lys193, Phe195, Glu234, Met238.
ZINC000100822646	TCM	−11.4	Thr112 (2.67), Tyr228 (2.99), Ser229 (2.92), Thr230 (2.74; 3.18).	Leu53, Phe83, Ser106, Leu108, Asp111, Phe113, Ala156, Leu158, Phe201, Ser203, Tyr204, Gly205.
NANPDB5642	Afro	−11.2	Asp111 (2.88), Tyr204 (2.86), Gly205 (3.07).	Leu53, Ser106, Leu108, Thr112, Phe113, Leu158, Phe201, Ser203, Tyr228, Ser229, Thr230.
ZINC000095486235	Afro	−11.1	Gly91 (2.98).	Cys42, Arg45, Arg98, Asp100, Tyr135, Tyr148, Ser185, Lys193, Phe195, Met238.
NANPDB2874	Afro	−11.1	Arg45 (3.02; 3.33), Gly91 (3.25), Tyr135 (2.81), Lys181 (3.26).	Asp100, Tyr136, Val139, Pro147, Tyr148, Ser185, Phe195.
ZINC000035941652	Afro	−11	Tyr135 (3.03), Lys181 (3.17), Ser185 (3.09), Glu234 (3.28).	Arg45, Arg98, Tyr136, Asp137, Val139, Pro147, Tyr148, Phe195.
NANPDB4566	Afro	−11	Tyr135 (2.9), Lys181 (2.8).	Arg45, Arg98, Asp100, Tyr136, Asp137, Val138, Val139, Ile146, Pro147, Tyr148, Ser185, Phe195, Glu234.
NANPDB513	Afro	−11	Glu114 (2.88), Ala116 (3.24), Thr115 (3.23), Tyr204 (3.07), Thr230 (2.95).	Leu53, Ser106, Thr112, Phe113, Leu158, Phe201, Ser203, Gly205, Tyr228, Ser229.
Acetylaleuritolic acid	Oncho	−10.3	Glu114 (3.18), Thr115 (2.93; 2.93), Ala116 (3.18), Tyr204 (2.81), Gly205 (3.1), Ser229 (2.8).	Phe83, Ser106, Leu108, Asp111, Thr112, Phe113, Glu114, Thr115, Ala116, Ser203, Tyr204, Gly205, Tyr228, Ser229, Thr230.
Rhemannic acid	Oncho	−9.5	Ser106 (3.09).	Ser106, Leu108, Asp111, Thr112, Phe113, Glu114, Ala116, Ala127, Leu158, Phe201, Tyr204, Tyr228, Ser229, Thr230.
Polycarpol	Oncho	−9.2	Ser106 (2.7), Thr112 (3.0).	Leu53, Thr112, Phe113, Ala116, Phe201, Ser106, Ala127, Phe201, Ser203, Tyr204, Tyr228, Ser229, Thr230.

**Table 4:** Binding free energies and the other contributing energies of the WSP-ligand complexes from MMPBSA computation. Energy values are presented as average ± standard deviations in kJ/mol.

COMPOUND	van der Waal Energy (kJ/mol)	Electrostatic Energy (kJ/mol)	Polar Solvation Energy (kJ/mol)	SASA Energy (kJ/mol)	Binding Energy (kJ/mol)
ZINC000095913861	−216.596 ± 30.529	−12.103 ± 11.973	141.841 ± 28.188	−24.215 ± 2.114	−111.073 ± 21.975
NANPDB4566	−158.603 ± 13.923	−45.067 ± 13.751	128.456 ± 20.752	−16.468 ± 1.345	−91.683 ± 14.928
ZINC000095486235	−131.255 ± 26.735	−3.563 ± 17.035	102.711 ± 38.032	−16.791 ± 2.507	−48.899 ± 18.047
ZINC000035941652	−148.894 ± 13.295	−43.213 ± 10.456	126.310 ± 17.926	−16.680 ± 0.994	−82.477 ± 16.348
Acetylaleuritolic acid	−137.347 ± 14.636	−2.452 ± 10.662	65.570 ± 18.150	−18.239 ± 1.759	−92.468 ± 15.900
Rhemannic acid	−152.808 ± 23.657	−25.762 ± 10.074	112.448 ± 26.331	−17.763 ±2.427	−83.884 ± 23.675

**Table 5:** A list of the selected compounds with their 2D structures and common/IUPAC names. The UPAC names were generated using MarvinSketch (ChemAxon Ltd., MarvinSketch version 17.17.0, Budapest, Hungary).

Compound	Common/Iupac Name	2d Structure
ZINC000095913861	(2E)-2,11,28-trimethyl-19-methylidene-13,30-dioxaheptacyclo [21.10.1.0^6,18^.0^7,15^.0^10,14^.0^24,32^.0^27,31^]tetratriaconta-1(33),2,6(18),7(15),10(14),11,16,23(34),24(32),27(31),28-undecaene-8,9,25,26-tetrone	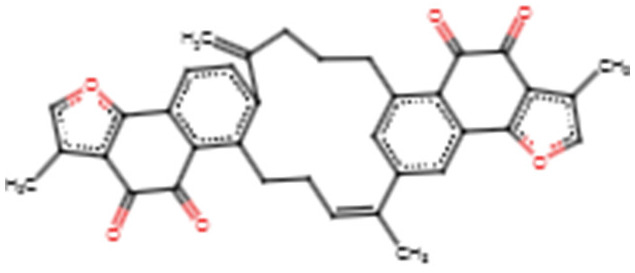
ZINC000095486235	(1S,13S,16R,18S)-12-[2-(4-hydroxyphenyl)ethyl]-18-methoxy-15-methyl-5,7-dioxa-12,15-diazapentacyclo[1 1.7.0.0^1,16^.0^2,10^.0^4,8^]icosa-2,4(8),9,19-tetraen-14-one	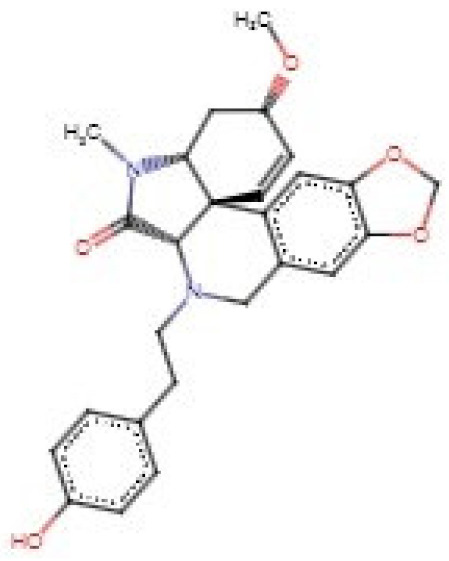
ZINC000035941652	(2S)-2-(2,2-dimethyl-3,4-dihydro-2H-1-benzopyran-6-yl)-7-hydroxy-3,4-dihydro-2H-1-benzopyran-4-one	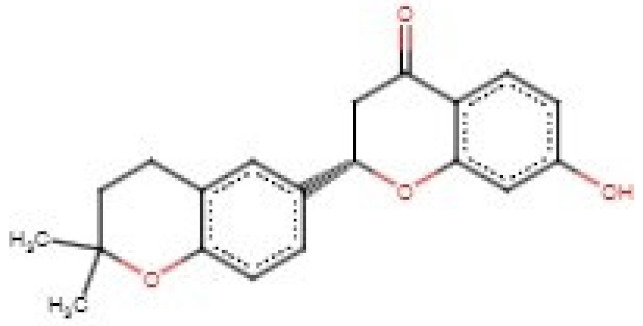
NANPDB4566	Coladonin	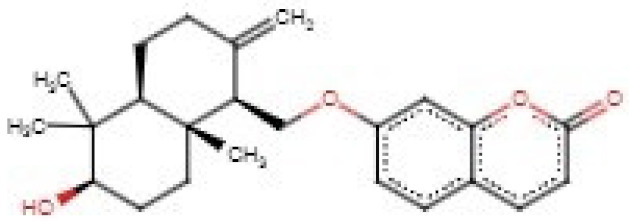
Acetylaleuritolic acid	Maprounic Acid Acetate or 3-acetylaleuritolic acid	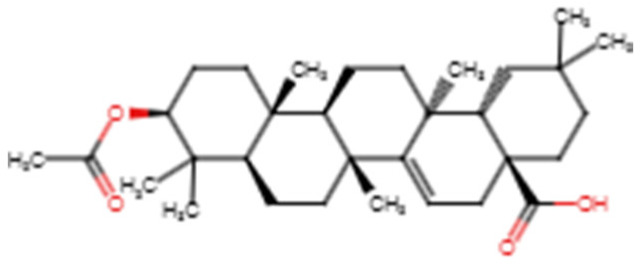
Rhemannic acid	Lantedene A	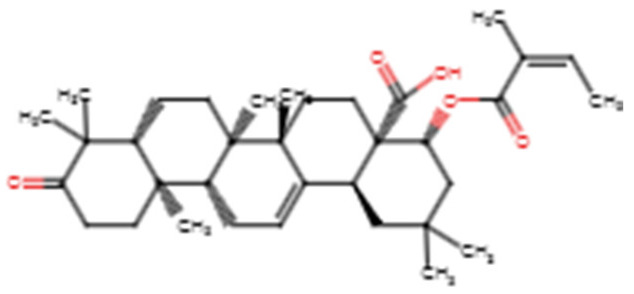

## Data Availability

Not applicable.

## Supplementary Materials

The following are available online at https://www.mdpi.com/article/10.3390/biomedicines9111682/s1, Figure S1: Cartoon representation of the top three predicted tertiary structures of the Wolbachia surface protein from the three techniques used: (A) MOD2 (B) ITAS1 and (C) ROB2. ITAS1 is in magenta, ROB2 in cyan and MOD2 in green, Figure S2: ERRAT plot for the WSP model. Red bars identify the misfolded region located distantly from the active site, yellow bars demonstrate the error region between 95% and 99%, and green bars indicate the region with a lower error rate for protein folding, Figure S3: Ramachandran plots of the protein structures obtained via PROCHECK. (A) Ramachandran plot of protein model MOD2 and (B) Ramachandran plot of protein model ITAS1. The percentages of residues of model MOD2 in the most favored regions, additionally allowed regions, generously allowed regions and disallowed regions are 82.5, 16.5, 1.0 and 0.0%, respectively. For model ITAS1, 53.9, 40.3, 3.9 and 1.9% of the amino acid residues were predicted to be in the most favored, additionally allowed, generously allowed and disallowed regions, respectively, Figure S4: Protein quality evaluation using ProSA. (A) ProSA-web z-score of the WSP indicating the overall model quality. (B) ProSA-web local model quality of the WSP by plotting energies as a function of amino acid sequence position, Figure S5: Predicted binding sites of the WSP (ROB2 model). (A) Binding site positions are shown with respect to conservation; (B) Superimposition of WSP (ROB2 model) and 1P4T (template structure) showing conserved binding site region in red and unconserved sites in blue; and (C) Ligands docked into the binding sites of the WSP. In (C), the protein is shown as a grey surface representation while pockets 1, 2, 3, 4, 5 and 6 are colored red, green, yellow, blue, hotpink and cyan, respectively, Figure S6: Cartoon representation of the WSP in complex with (A) NANPDB5642, (B) ZINC000095486235 and (C) acetylaleuritolic acid. The binding sites are represented as surface and the ligands are shown as sticks, Figure S7: Two-dimensional plots of the WSP-ligand interactions generated using LigPlot+. Interaction profiles of (A) WSP- ZINC000095486235 complex and (B) WSP-Acetylaleuritolic acid complex. Hydrophobic contacts are represented as red spoke arcs, hydrogen bonds are represented as green dash lines and the ligands are colored purple, Figure S8: Molecular mechanics/Poisson-Boltzmann surface area (MMPBSA) plots showing the binding free energy contribution per residue of the (A) WSP-ZINC000095486235, (B) WSP-ZINC000035941652, (C) WSP-ZINC000095913861, (D) WSP-acetylaleuritolic acid and (E) WSP-rhemannic acid complexes, Table S1: The top five protein structure templates predicted via SWISS-MODEL, Table S2: The DOPE and GA341 scores of the five models predicted using Modeller, Table S3: The C-Scores of the five models predicted by I-TASSER, Table S4: Structural Evaluation of the Robetta Generated Models using SAVES v5.0, Table S5: Evaluation of the top three WSP models generated using Robetta, I-TASSER and Modeller. Models ROB2, ITAS1 and MOD2 were generated using Robetta, I-TASSER and Modeller, respectively, Table S6: ADME Prediction of some compounds for Gastrointestinal (GI); Blood Brain Barrier (BBB); Estimated Solubility (ESOL) class, P-glycoprotein (Pgp) and TPSA, Table S7: Toxicological profiles of some selected compounds, Table S8: Binding energies of the top **20** non-redundant lead compounds each of the TCM and Afro libraries and the top **11** onchocerca spp compounds against the three top models (ROB2, ITAS1 and MOD2), Table S9: Previously modelled WSP structures and the templates used.

## References

[1] Rodríguez-Pérez M.A., Unnasch T.R., Real-Najarro O. (2011). Assessment and Monitoring of Onchocerciasis in Latin America. Advances in Parasitology.

[2] Ae-Ngibise K., Akpalu B., Ngugi A.K., Akpalu A., Agbokey F., Adjei P., Punguyire D., Bottomley C., Newton C.R., Owusu-Agyei S. (2015). Prevalence and risk factors for Active Convulsive Epilepsy in Kintampo, Ghana. Pan Afr. Med. J..

[3] Bakowski M.A., McNamara C.W. (2019). Advances in Antiwolbachial Drug Discovery for Treatment of Parasitic Filarial Worm Infections. Trop. Med. Infect. Dis..

[4] Basáñez M.G., Pion S.D.S., Churcher T.S., Breitling L.P., Little M.P., Boussinesq M. (2006). River blindness: A success story under threat?. PLoS Med..

[5] Hotez P.J., Kamath A. (2009). Neglected tropical diseases in sub-Saharan Africa: Review of their prevalence, distribution, and disease burden. PLoS Negl. Trop. Dis..

[6] Seymour J., Kinder M., Benton B.B. (2007). Case 7: Controlling Onchocerciasis (River Blindness) in Sub-Saharan Africa. Case Studies in Global Health: Millions Saved.

[7] Colebunders R., Njamnshi A.K., Menon S., Newton C.R., Hotterbeekx A., Preux P.-M., Hopkins A., Vaillant M., Siewe Fodjo J.N. (2021). Onchocerca volvulus and epilepsy: A comprehensive review using the Bradford Hill criteria for causation. PLoS Negl. Trop. Dis..

[8] Hotterbeekx A., Raimon S., Abd-Elfarag G., Carter J.Y., Sebit W., Suliman A., Siewe Fodjo J.N., De Witte P., Logora M.Y., Colebunders R. (2020). Onchocerca volvulus is not detected in the cerebrospinal fluid of persons with onchocerciasis-associated epilepsy. Int. J. Infect. Dis..

[9] Abegunde A.T., Ahuja R.M., Okafor N.J. (2016). Doxycycline plus ivermectin versus ivermectin alone for treatment of patients with onchocerciasis. Cochrane Database Syst. Rev..

[10] Coffeng L.E., Stolk W.A., Zouré H.G.M., Veerman J.L., Agblewonu K.B., Murdoch M.E., Noma M., Fobi G., Richardus J.H., Bundy D.A.P. (2013). African Programme for Onchocerciasis Control 1995–2015: Model-Estimated Health Impact and Cost. PLoS Negl. Trop. Dis..

[11] Taylor M.J., Hoerauf A., Bockarie M. (2010). Lymphatic filariasis and onchocerciasis. Lancet.

[12] Hoerauf A., Mand S., Adjei O., Fleischer B., Büttner D.W. (2001). Depletion of wolbachia endobacteria in Onchocerca volvulus by doxycycline and microfilaridermia after ivermectin treatment. Lancet.

[13] Hopkins A.D. (2005). Ivermectin and onchocerciasis: Is it all solved?. Eye.

[14] Enk C.D. (2006). Onchocerciasis—river blindness. Clin. Dermatol..

[15] Osei-Atweneboana M.Y., Awadzi K., Attah S.K., Boakye D.A., Gyapong J.O., Prichard R.K. (2011). Phenotypic evidence of emerging ivermectin resistance in Onchocerca volvulus. PLoS Negl. Trop. Dis..

[16] Osei-Atweneboana M.Y., Eng J.K., Boakye D.A., Gyapong J.O., Prichard R.K. (2007). Prevalence and intensity of Onchocerca volvulus infection and efficacy of ivermectin in endemic communities in Ghana: A two-phase epidemiological study. Lancet.

[17] Debrah A.Y., Specht S., Klarmann-Schulz U., Batsa L., Mand S., Marfo-Debrekyei Y., Fimmers R., Dubben B., Kwarteng A., Osei-Atweneboana M. (2015). Doxycycline Leads to Sterility and Enhanced Killing of Female Onchocerca volvulus Worms in an Area with Persistent Microfilaridermia after Repeated Ivermectin Treatment: A Randomized, Placebo-Controlled, Double-Blind Trial. Clin. Infect. Dis..

[18] Frempong K.K., Walker M., Cheke R.A., Tetevi E.J., Gyan E.T., Owusu E.O., Wilson M.D., Boakye D.A., Taylor M.J., Biritwum N.K. (2016). Does increasing treatment frequency address suboptimal responses to ivermectin for the control and elimination of river blindness?. Clin. Infect. Dis..

[19] Ali M.M.M., Mukhtar M.M., Baraka O.Z., Homeida M.M.A., Kheir M.M., Mackenzie C.D. (2002). Immunocompetence may be important in the effectiveness of Mectizan® (ivermectin) in the treatment of human onchocerciasis. Acta Trop..

[20] Awadzi K., Boakye D.A., Edwards G., Opoku N.O., Attah S.K., Osei-Atweneboana M.Y., Lazdins-Helds J.K., Ardrey A.E., Addy E.T., Quartey B.T. (2004). An investigation of persistent microfilaridermias despite multiple treatments with ivermectin, in two onchocerciasis-endemic foci in Ghana. Ann. Trop. Med. Parasitol..

[21] Kuesel A.C. (2016). Research for new drugs for elimination of onchocerciasis in Africa. Int. J. Parasitol. Drugs Drug Resist..

[22] Jawahar S., Tricoche N., Bulman C.A., Sakanari J., Lustigman S. (2021). Drugs that target early stages of Onchocerca volvulus: A revisited means to facilitate the elimination goals for onchocerciasis. PLoS Negl. Trop. Dis..

[23] Buchter V., Hofmann D., Häberli C., Keiser J. (2021). Characterization of Moxidectin against Strongyloides ratti: In vitro and in vivo activity and pharmacokinetics in the rat model. ACS Infect. Dis..

[24] Jolodar A., Fischer P., Bergmann S., Bu D.W., Hammerschmidt S., Brattig N.W. (2003). Molecular cloning of an a -enolase from the human filarial parasite Onchocerca volvulus that binds human plasminogen. Biochim. Et Biophys. Acta BBA Gene Struct. Expr..

[25] Bakowski M.A., Shiroodi R.K., Liu R., Olejniczak J., Yang B., Gagaring K., Guo H., White P.M., Chappell L., Debec A. (2019). Discovery of short-course antiwolbachial quinazolines for elimination of filarial worm infections. Sci. Transl. Med..

[26] Uday J., Puttaraju H.P. (2012). Comparative analysis of Wolbachia surface protein in D. melanoagster, A. tabida and B. malayi. Bioinformation.

[27] Tamarozzi F., Halliday A., Gentil K., Hoerauf A., Pearlman E., Taylor M.J. (2011). Onchocerciasis: The role of Wolbachia bacterial endosymbionts in parasite biology, disease pathogenesis, and treatment. Clin. Microbiol. Rev..

[28] Gillette-Ferguson I., Hise A.G., McGarry H.F., Turner J., Esposito A., Sun Y., Diaconu E., Taylor M.J., Pearlman E. (2004). Wolbachia -Induced Neutrophil Activation in a Mouse Model of Ocular Onchocerciasis (River Blindness). Infect. Immun..

[29] Bandi C., Trees A.J., Brattig N.W. (2001). Wolbachia in filarial nematodes: Evolutionary aspects and implications for the pathogenesis and treatment of filarial diseases. Vet. Parasitol..

[30] Shiny C., Krushna N.S.A., Haripriya K., Babu S., Elango S., Manokaran G., Narayanan R.B. (2012). Recombinant Wolbachia surface protein (WSP)-induced T cell responses in Wuchereria bancrofti infections. Parasitol. Res..

[31] Braig H.R., Zhou W., Dobson S.L., O’Neill S.L. (1998). Cloning and characterization of a gene encoding the major surface protein of the bacterial endosymbiont Wolbachia pipientis. J. Bacteriol..

[32] Bazzocchi C., Jamnongluk W., O’Neill S.L., Anderson T.J.C., Genchi C., Bandi C. (2000). wsp Gene sequences from the Wolbachia of filarial nematodes. Curr. Microbiol..

[33] Jiggins F.M., Hurst G.D.D., Yang Z. (2002). Host-symbiont conflicts: Positive selection on an outer membrane protein of parasitic but not mutualistic Rickettsiaceae. Mol. Biol. Evol..

[34] Ottesen E.A. (1995). Immune responsiveness and the pathogenesis of human onchocerciasis. J. Infect. Dis..

[35] Hoerauf A., Brattig N. (2002). Resistance and susceptibility in human onchocerciasis–Beyond Th1 vs Th2. Trends Parasitol..

[36] Brattig N.W., Bazzocchi C., Kirschning C.J., Reiling N., Büttner D.W., Ceciliani F., Geisinger F., Hochrein H., Ernst M., Wagner H. (2004). The major surface protein of Wolbachia endosymbionts in filarial nematodes elicits immune responses through TLR2 and TLR4. J. Immunol..

[37] Melnikow E., Xu S., Liu J., Bell A.J., Ghedin E., Unnasch T.R., Lustigman S. (2013). A Potential Role for the Interaction of Wolbachia Surface Proteins with the Brugia malayi Glycolytic Enzymes and Cytoskeleton in Maintenance of Endosymbiosis. PLoS Negl. Trop. Dis..

[38] Voronin D., Bachu S., Shlossman M., Unnasch T.R., Ghedin E., Lustigman S. (2016). Glucose and Glycogen Metabolism in Brugia malayi Is Associated with Wolbachia Symbiont Fitness. PLoS ONE.

[39] Giombini E., Orsini M., Carrabino D., Tramontano A. (2010). An automatic method for identifying surface proteins in bacteria: SLEP. BMC Bioinform..

[40] Maione D. (2005). Identification of a Universal Group B Streptococcus Vaccine by Multiple Genome Screen. Science.

[41] Lindahl G., Stålhammar-Carlemalm M., Areschoug T. (2005). Surface Proteins of Streptococcus agalactiae and Related Proteins in Other Bacterial Pathogens. Clin. Microbiol. Rev..

[42] Johnston K.L., Taylor M.J. (2007). Wolbachia in filarial parasites: Targets for filarial infection and disease control. Curr. Infect. Dis. Rep..

[43] Langworthy N.G., Renz A., Mackenstedt U., Henkle-Dührsen K., de Bronsvoort M.B., Tanya V.N., Donnelly M.J., Trees A.J. (2000). Macrofilaricidal activity of tetracycline against the filarial nematode Onchocerca ochengi: Elimination of Wolbachia precedes worm death and suggests a dependent relationship. Proc. Biol. Sci..

[44] Townson S., Tagboto S., McGarry H.F., Egerton G.L., Taylor M.J. (2006). Onchocerca parasites and Wolbachia endosymbionts: Evaluation of a spectrum of antibiotic types for activity against Onchocerca gutturosa in vitro. Filaria J..

[45] Supali T., Djuardi Y., Pfarr K.M., Wibowo H., Taylor M.J., Hoerauf A., Houwing-Duistermaat J.J., Yazdanbakhsh M., Sartono E. (2008). Doxycycline Treatment of *Brugia malayi*–Infected Persons Reduces Microfilaremia and Adverse Reactions after Diethylcarbamazine and Albendazole Treatment. Clin. Infect. Dis..

[46] Büttner D.W., Wanji S., Bazzocchi C., Bain O., Fischer P. (2003). Obligatory symbiotic Wolbachia endobacteria are absent from Loa loa. Filaria J..

[47] Klarmann-Schulz U., Specht S., Debrah A.Y., Batsa L., Ayisi-Boateng N.K., Osei-Mensah J., Mubarik Y., Konadu P., Ricchiuto A., Fimmers R. (2017). Comparison of Doxycycline, Minocycline, Doxycycline plus Albendazole and Albendazole Alone in Their Efficacy against Onchocerciasis in a Randomized, Open-Label, Pilot Trial. PLoS Negl. Trop. Dis..

[48] Ventola C.L. (2015). The Antibiotic Resistance Crisis. Pharm. Ther..

[49] Segura-Cabrera A., Bocanegra-García V., Lizarazo-Ortega C., Guo X., Correa-Basurto J., Rodríguez-Pérez M.A. (2011). A computational analysis of the binding mode of closantel as inhibitor of the Onchocerca volvulus chitinase: Insights on macrofilaricidal drug design. J. Comput. Aided. Mol. Des..

[50] Vildina J.D., Kalmobe J., Djafsia B., Schmidt T.J., Liebau E., Ndjonka D. (2017). A Hydro-Alcoholic Extract from the Fruits of Acacia nilotica and Some Proanthocyanidin Derivatives. Molecules.

[51] Broni E., Adoboe D., Nsoh J., Yunus U.F., Kwofie K.S. Computational Drug Design: Identifying Potential Drugs for Onchocerciasis via Wolbachia Surface Protein. Proceedings of the Ghana Biomedical Convention.

[52] Cragg G.M., Newman D.J. (2013). Natural products: A continuing source of novel drug leads. Biochim. Biophys. Acta Gen. Subj..

[53] Thompson K.D. (2006). Lead Molecules from Natural Products–Discovery and New Trends. Adv. Phytomed..

[54] Lagunin A., Stepanchikova A., Filimonov D., Poroikov V. (2000). PASS: Prediction of activity spectra for biologically active substances. Bioinformatics.

[55] Poroikov V.V., Filimonov D.A., Ihlenfeldt W.-D., Gloriozova T.A., Lagunin A.A., Borodina Y.V., Stepanchikova A.V., Nicklaus M.C. (2003). PASS Biological Activity Spectrum Predictions in the Enhanced Open NCI Database Browser. J. Chem. Inf. Comput. Sci..

[56] Parasuraman S. (2011). Prediction of activity spectra for substances. J. Pharmacol. Pharmacother..

[57] Kiefer F., Arnold K., Künzli M., Bordoli L., Schwede T. (2009). The SWISS-MODEL Repository and associated resources. Nucleic Acids Res..

[58] Bienert S., Waterhouse A., De Beer T.A.P., Tauriello G., Studer G., Bordoli L., Schwede T. (2017). The SWISS-MODEL Repository-new features and functionality. Nucleic Acids Res..

[59] Muhammed M.T., Aki-Yalcin E. (2019). Homology modeling in drug discovery: Overview, current applications, and future perspectives. Chem. Biol. Drug Des..

[60] Dolan M.A., Noah J.W., Hurt D. (2012). Comparison of common homology modeling algorithms: Application of user-defined alignments. Methods Mol. Biol..

[61] Nayeem A. (2006). A comparative study of available software for high-accuracy homology modeling: From sequence alignments to structural models. Protein Sci..

[62] Broni E., Kwofie S.K., Asiedu S.O., Miller W.A., Wilson M.D. (2021). A molecular modeling approach to identify potential antileishmanial compounds against the cell division cycle (Cdc)-2-related kinase 12 (crk12) receptor of leishmania donovani. Biomolecules.

[63] Kuntal B.K., Aparoy P., Reddanna P. (2010). EasyModeller: A graphical interface to MODELLER. BMC Res. Notes.

[64] Yang J., Zhang Y. (2015). I-TASSER server: New development for protein structure and function predictions. Nucleic Acids Res..

[65] Zhang Y. (2008). I-TASSER server for protein 3D structure prediction. BMC Bioinform..

[66] Roy A., Kucukural A., Zhang Y. (2010). I-TASSER: A unified platform for automated protein structure and function prediction. Nat. Protoc..

[67] Yang J., Yan R., Roy A., Xu D., Poisson J., Zhang Y. (2014). The I-TASSER Suite: Protein structure and function prediction. Nat. Methods.

[68] Song Y., Dimaio F., Wang R.Y.R., Kim D., Miles C., Brunette T., Thompson J., Baker D. (2013). High-resolution comparative modeling with RosettaCM. Structure.

[69] Raman S., Vernon R., Thompson J., Tyka M., Sadreyev R., Pei J., Kim D., Kellogg E., Dimaio F., Lange O. (2009). Structure prediction for CASP8 with all-atom refinement using Rosetta. Proteins Struct. Funct. Bioinform..

[70] Xu D., Zhang Y. (2011). Improving the physical realism and structural accuracy of protein models by a two-step atomic-level energy minimization. Biophys. J..

[71] Wang Q., Canutescu A.A., Dunbrack R.L. (2008). SCWRL and MolIDE: Computer programs for side-chain conformation prediction and homology modeling. Nat. Protoc..

[72] Krivov G.G., Shapovalov M.V., Dunbrack R.L. (2009). Improved prediction of protein side-chain conformations with SCWRL4. Proteins Struct. Funct. Bioinform..

[73] Colovos C., Yeates T.O. (1993). Verification of protein structures: Patterns of nonbonded atomic interactions. Protein Sci..

[74] Pontius J., Richelle J., Wodak S.J. (1996). Deviations from Standard Atomic Volumes as a Quality Measure for Protein Crystal Structures. J. Mol. Biol..

[75] Bowie J., Luthy R., Eisenberg D. (1991). A method to identify protein sequences that fold into a known three-dimensional structure. Science.

[76] Lüthy R., Bowie J.U., Eisenberg D. (1992). Assessment of protein models with three-dimensional profiles. Nature.

[77] Laskowski R.A., MacArthur M.W., Moss D.S., Thornton J.M. (1993). PROCHECK: A program to check the stereochemical quality of protein structures. J. Appl. Crystallogr..

[78] Ntie-Kang F., Zofou D., Babiaka S.B., Meudom R., Scharfe M., Lifongo L.L., Mbah J.A., Mbaze L.M., Sippl W., Efange S.M.N. (2013). AfroDb: A Select Highly Potent and Diverse Natural Product Library from African Medicinal Plants. PLoS ONE.

[79] Sterling T., Irwin J.J. (2015). ZINC 15–Ligand Discovery for Everyone. J. Chem. Inf. Model..

[80] Ntie-Kang F., Telukunta K.K., Döring K., Simoben C.V., Moumbock A.F.A., Malange Y.I., Njume L.E., Yong J.N., Sippl W., Günther S. (2017). NANPDB: A Resource for Natural Products from Northern African Sources. J. Nat. Prod..

[81] Chen C.Y.-C.C. (2011). TCM Database@Taiwan: The world’s largest traditional Chinese medicine database for drug screening In Silico. PLoS ONE.

[82] Binkowski T.A. (2003). CASTp: Computed Atlas of Surface Topography of proteins. Nucleic Acids Res..

[83] Dundas J., Ouyang Z., Tseng J., Binkowski A., Turpaz Y., Liang J. (2006). CASTp: Computed atlas of surface topography of proteins with structural and topographical mapping of functionally annotated residues. Nucleic Acids Res..

[84] Sander T., Freyss J., Von Korff M., Rufener C. (2015). DataWarrior: An open-source program for chemistry aware data visualization and analysis. J. Chem. Inf. Model..

[85] Dallakyan S., Olson A.J. (2015). Small-Molecule Library Screening by Docking with PyRx. Methods in Molecular Biology.

[86] O’Boyle N.M., Banck M., James C.A., Morley C., Vandermeersch T., Hutchison G.R. (2011). Open Babel: An open chemical toolbox. J. Cheminform..

[87] Trott O., Olson A.J. (2010). AutoDock Vina: Improving the Speed and Accuracy of Docking with a New Scoring Function, EfficientOptimization, and Multithreading. J. Comput. Chem..

[88] Rother K. (2005). Introduction to PyMOL. Methods Mol. Biol. Clift. Nj.

[89] Yuan S., Chan H.C.S., Hu Z. (2017). Using PyMOL as a platform for computational drug design. Wiley Interdiscip. Rev. Comput. Mol. Sci..

[90] Kairys V., Fernandes M.X., Gilson M.K. (2006). Screening Drug-Like Compounds by Docking to Homology Models: A Systematic Study. J. Chem. Inf. Model..

[91] Daina A., Michielin O., Zoete V. (2017). SwissADME: A free web tool to evaluate pharmacokinetics, drug-likeness and medicinal chemistry friendliness of small molecules. Sci. Rep..

[92] Laskowski R.A., Swindells M.B. (2011). LigPlot+: Multiple Ligand–Protein Interaction Diagrams for Drug Discovery. J. Chem. Inf. Model..

[93] Kumari R., Kumar R., Lynn A. (2014). g_mmpbsa —A GROMACS Tool for High-Throughput MM-PBSA Calculations. J. Chem. Inf. Model..

[94] Campanera J.M., Pouplana R. (2010). MMPBSA Decomposition of the Binding Energy throughout a Molecular Dynamics Simulation of Amyloid-Beta (Aß10−35) Aggregation. Molecules.

[95] Wang C., Greene D., Xiao L., Qi R., Luo R. (2018). Recent Developments and Applications of the MMPBSA Method. Front. Mol. Biosci..

[96] Gasteiger E., Hoogland C., Gattiker A., Duvaud S., Wilkins M.R., Appel R.D., Bairoch A. (2005). Protein Identification and Analysis Tools on the ExPASy Server. The Proteomics Protocols Handbook.

[97] Gamage D.G., Gunaratne A., Periyannan G.R., Russell T.G. (2019). Applicability of Instability Index for In vitro Protein Stability Prediction. Protein Pept. Lett..

[98] Vandeputte-Rutten L., Bos M.P., Tommassen J., Gros P. (2003). Crystal Structure of Neisserial Surface Protein A (NspA), a Conserved Outer Membrane Protein with Vaccine Potential. J. Biol. Chem..

[99] Larsson P., Wallner B., Lindahl E., Elofsson A. (2008). Using multiple templates to improve quality of homology models in automated homology modeling. Protein Sci..

[100] Fox D.A., Larsson P., Lo R.H., Kroncke B.M., Kasson P.M., Columbus L. (2014). Structure of the neisserial outer membrane protein Opa60: Loop flexibility essential to receptor recognition and bacterial engulfment. J. Am. Chem. Soc..

[101] Melo F., Sánchez R., Sali A. (2009). Statistical potentials for fold assessment. Protein Sci..

[102] Eswar N., Webb B., Marti-Renom M.A., Madhusudhan M.S., Eramian D., Shen M., Pieper U., Sali A. (2006). Comparative Protein Structure Modeling Using Modeller. Curr. Protoc. Bioinforma..

[103] Shen M., Sali A. (2006). Statistical potential for assessment and prediction of protein structures. Protein Sci..

[104] Renault M., Saurel O., Czaplicki J., Demange P., Gervais V., Löhr F., Réat V., Piotto M., Milon A. (2009). Solution State NMR Structure and Dynamics of KpOmpA, a 210 Residue Transmembrane Domain Possessing a High Potential for Immunological Applications. J. Mol. Biol..

[105] Mora Lagares L., Minovski N., Caballero Alfonso A.Y., Benfenati E., Wellens S., Culot M., Gosselet F., Novič M. (2020). Homology Modeling of the Human P-glycoprotein (ABCB1) and Insights into Ligand Binding through Molecular Docking Studies. Int. J. Mol. Sci..

[106] Hoda S., Gupta L., Shankar J., Gupta A.K., Vijayaraghavan P. (2020). cis-9-Hexadecenal, a Natural Compound Targeting Cell Wall Organization, Critical Growth Factor, and Virulence of Aspergillus fumigatus. ACS Omega.

[107] Wiederstein M., Sippl M.J. (2007). ProSA-web: Interactive web service for the recognition of errors in three-dimensional structures of proteins. Nucleic Acids Res..

[108] Sippl M.J. (1993). Recognition of errors in three-dimensional structures of proteins. Proteins Struct. Funct. Genet..

[109] Tian W., Chen C., Lei X., Zhao J., Liang J. (2018). CASTp 3.0: Computed atlas of surface topography of proteins. Nucleic Acids Res..

[110] Kwofie S.K., Broni E., Teye J., Quansah E., Issah I., Wilson M.D., Miller W.A., Tiburu E.K., Bonney J.H.K. (2019). Pharmacoinformatics-based identification of potential bioactive compounds against Ebola virus protein VP24. Comput. Biol. Med..

[111] Kwofie S.K., Broni E., Asiedu S.O., Kwarko G.B., Dankwa B., Enninful K.S., Tiburu E.K., Wilson M.D. (2021). Cheminformatics-Based Identification of Potential Novel Anti-SARS-CoV-2 Natural Compounds of African Origin. Molecules.

[112] Konc J., Janezic D. (2012). ProBiS-2012: Web server and web services for detection of structurally similar binding sites in proteins. Nucleic Acids Res..

[113] Pautsch A., Schulz G.E. (2000). High-resolution structure of the OmpA membrane domain. J. Mol. Biol..

[114] Pascal T.A., Abrol R., Mittal R., Wang Y., Prasadarao N.V., Goddard W.A. (2010). Experimental Validation of the Predicted Binding Site of Escherichia coli K1 Outer Membrane Protein A to Human Brain Microvascular Endothelial Cells. J. Biol. Chem..

[115] Samje M., Metuge J., Mbah J., Nguesson B., Cho-Ngwa F. (2014). In vitro anti-Onchocerca ochengi activities of extracts and chromatographic fractions of Craterispermum laurinum and Morinda lucida. BMC Complement. Altern. Med..

[116] Owolabi M.S., Padilla-Camberos E., Ogundajo A.L., Ogunwande I.A., Flamini G., Yusuff O.K., Allen K., Flores-Fernandez K.I., Flores-Fernandez J.M. (2014). Insecticidal activity and chemical composition of the morinda lucida essential oil against pulse beetle callosobruchus maculatus. Sci. World J..

[117] Metuge J.A., Nyongbela K.D., Mbah J.A., Samje M., Fotso G., Babiaka S.B., Cho-Ngwa F. (2014). Anti-Onchocerca activity and phytochemical analysis of an essential oil from Cyperus articulatus L.. BMC Complement. Altern. Med..

[118] Hassanein H.D., Nazif N.M., Shahat A.A., Hammouda F.M., Aboutable E.S.A., Saleh M.A. (2014). Chemical Diversity of Essential Oils from Cyperus articulatus, Cyperus esculentus and Cyperus papyrus. J. Essent. Oil Bearing Plants.

[119] Olawore N.O., Usman L.A., Ogunwande I.A., Adeleke K.A. (2006). Constituents of Rhizome Essential Oils of Two Types of Cyperus articulatus L. Grown in Nigeria. J. Essent. Oil Res..

[120] Smith B.B., James A.M., Fidelis C.N., Jonathan A.M., Simon M.N.E. (2016). Isolation and characterization of filaricidal compounds from the stem bark of Voacanga africana, a plant used in the traditional treatment of onchocerciasis in Cameroon. J. Med. Plants Res..

[121] Ngwewondo A., Wang M., Manfo F.P.T., Samje M., Ganin’s J.N., Ndi E., Andersen R.J., Cho-Ngwa F. (2018). Filaricidal properties of Lantana camara and Tamarindus indica extracts, and Lantadene A from L. camara against Onchocerca ochengi and Loa loa. PLoS Negl. Trop. Dis..

[122] Ndjonka D., Abladam E.D., Djafsia B., Ajonina-Ekoti I., Achukwi M.D., Liebau E. (2014). Anthelmintic activity of phenolic acids from the axlewood tree Anogeissus leiocarpus on the filarial nematode Onchocerca ochengi and drug-resistant strains of the free-living nematode Caenorhabditis elegans. J. Helminthol..

[123] Nyasse B., Ngantchou I., Nono J.J., Schneider B. (2006). Antifilarial activity in vitro of polycarpol and 3-*O*-acetyl aleuritolic acid from cameroonian medicinal plants against Onchocerca gutturosa. Nat. Prod. Res..

[124] Wang Y., Xiao J., Suzek T.O., Zhang J., Wang J., Bryant S.H. (2009). PubChem: A public information system for analyzing bioactivities of small molecules. Nucleic Acids Res..

[125] Kim S., Chen J., Cheng T., Gindulyte A., He J., He S., Li Q., Shoemaker B.A., Thiessen P.A., Yu B. (2021). PubChem in 2021: New data content and improved web interfaces. Nucleic Acids Res..

[126] Kim S., Thiessen P.A., Bolton E.E., Chen J., Fu G., Gindulyte A., Han L., He J., He S., Shoemaker B.A. (2016). PubChem Substance and Compound databases. Nucleic Acids Res..

[127] Chang M.W., Lindstrom W., Olson A.J., Belew R.K. (2007). Analysis of HIV wild-type and mutant structures via in silico docking against diverse ligand libraries. J. Chem. Inf. Model..

[128] Sharma A., Tiwari V., Sowdhamini R. (2020). Computational search for potential COVID-19 drugs from FDA-approved drugs and small molecules of natural origin identifies several anti-virals and plant products. J. Biosci..

[129] Turner J.D., Sharma R., Al Jayoussi G., Tyrer H.E., Gamble J., Hayward L., Priestley R.S., Murphy E.A., Davies J., Waterhouse D. (2017). Albendazole and antibiotics synergize to deliver short-course anti- Wolbachia curative treatments in preclinical models of filariasis. Proc. Natl. Acad. Sci. USA.

[130] Dangi A., Dwivedi V., Vedi S., Owais M., Misra-Bhattacharya S. (2010). Improvement in the antifilarial efficacy of doxycycline and rifampicin by combination therapy and drug delivery approach. J. Drug Target..

[131] Specht S., Pfarr K.M., Arriens S., Hübner M.P., Klarmann-Schulz U., Koschel M., Sternberg S., Martin C., Ford L., Taylor M.J. (2018). Combinations of registered drugs reduce treatment times required to deplete Wolbachia in the Litomosoides sigmodontis mouse model. PLoS Negl. Trop. Dis..

[132] Wen C.C., Kuo Y.H., Jan J.T., Liang P.H., Wang S.Y., Liu H.G., Lee C.K., Chang S.T., Kuo C.J., Lee S.S. (2007). Specific plant terpenoids and lignoids possess potent antiviral activities against severe acute respiratory syndrome coronavirus. J. Med. Chem..

[133] Veber D.F., Johnson S.R., Cheng H., Smith B.R., Ward K.W., Kopple K.D. (2002). Molecular Properties That Influence the Oral Bioavailability of Drug Candidates. J. Med. Chem..

[134] Egieyeh S.A., Syce J., Malan S.F., Christoffels A. (2016). Prioritization of anti-malarial hits from nature: Chemo-informatic profiling of natural products with in vitro antiplasmodial activities and currently registered anti-malarial drugs. Malar. J..

[135] Ganesan A. (2008). The impact of natural products upon modern drug discovery. Curr. Opin. Chem. Biol..

[136] Van Breemen M.S.M., Wilms E.B., Vecht C.J. (2007). Epilepsy in patients with brain tumours: Epidemiology, mechanisms, and management. Lancet Neurol..

[137] Abd-Elfarag G., Carter J.Y., Raimon S., Sebit W., Suliman A., Fodjo J.N.S., Olore P.C., Biel K.P., Ojok M., Logora M.Y. (2020). Persons with onchocerciasis-associated epilepsy and nodding seizures have a more severe form of epilepsy with more cognitive impairment and higher levels of Onchocerca volvulus infection. Epileptic Disord..

[138] Callus B.A., Vaux D.L. (2007). Caspase inhibitors: Viral, cellular and chemical. Cell Death Differ..

[139] McIlwain D.R., Berger T., Mak T.W. (2013). Caspase functions in cell death and disease. Cold Spring Harb. Perspect. Biol..

[140] Pearlman E. (2004). Interleukin 4 and T helper type 2 cells are required for development of experimental onchocercal keratitis (river blindness). J. Exp. Med..

[141] Lange A.M., Yutanawiboonchai W., Scott P., Abraham D. (1994). IL-4- and IL-5-dependent protective immunity to Onchocerca volvulus infective larvae in BALB/cBYJ mice. J. Immunol..

[142] Kerepesi L.A., Leon O., Lustigman S., Abraham D. (2005). Protective immunity to the larval stages of Onchocerca volvulus is dependent on toll-like receptor 4. Infect. Immun..

[143] Annapareddy P. (2012). Identifying And Quantifying Flavonoids In Three Medicinal Plants By Hplc. Int. J. Innov. Res. Dev..

[144] Eguale T., Tadesse D., Giday M. (2011). In vitro anthelmintic activity of crude extracts of five medicinal plants against egg-hatching and larval development of Haemonchus contortus. J. Ethnopharmacol..

[145] Satpute S.M., Bhamburdekar S.B., Gaikwad D.K. (2017). Anthelmintic Potential of Pods and Stem Bark Extracts of Cassia Fistula L.. Int. Res. J. Pharm..

[146] Irshad M., Singh M., Rizvi M. (2010). Assessment of Anthelmintic Activity of Cassia fistula L.. Middle East J. Sci. Res..

[147] Bhalodia N.R., Shukla V.J. (2011). Antibacterial and antifungal activities from leaf extracts of Cassia fistula l.: An ethnomedicinal plant. J. Adv. Pharm. Technol. Res..

[148] Tamarozzi F., Turner J.D., Pionnier N., Midgley A., Guimaraes A.F., Johnston K.L., Edwards S.W., Taylor M.J. (2016). Wolbachia endosymbionts induce neutrophil extracellular trap formation in human onchocerciasis. Sci. Rep..

[149] Eng J.K.L., Blackhall W.J., Osei-Atweneboana M.Y., Bourguinat C., Galazzo D., Beech R.N., Unnasch T.R., Awadzi K., Lubega G.W., Prichard R.K. (2006). Ivermectin selection on β-tubulin: Evidence in Onchocerca volvulus and Haemonchus contortus. Mol. Biochem. Parasitol..

[150] Kushwaha S., Soni V.K., Singh P.K., Bano N., Kumar A., Sangwan R.S., Misra-Bhattacharya S. (2012). Withania somnifera chemotypes NMITLI 101R, NMITLI 118R, NMITLI 128R and withaferin A protect Mastomys coucha from Brugia malayi infection. Parasite Immunol..

[151] Dar N.J., Bhat J.A., Satti N.K., Sharma P.R., Hamid A., Ahmad M. (2017). Withanone, an Active Constituent from Withania somnifera, Affords Protection Against NMDA-Induced Excitotoxicity in Neuron-Like Cells. Mol. Neurobiol..

[152] Konar A., Shah N., Singh R., Saxena N., Kaul S.C., Wadhwa R., Thakur M.K. (2011). Protective role of Ashwagandha leaf extract and its component withanone on scopolamine-induced changes in the brain and brain-derived cells. PLoS ONE.

[153] Ragasa C.Y., Hofileña J.G., Rideout J.A. (2002). New furanoid diterpenes from Caesalpinia pulcherrima. J. Nat. Prod..

[154] Pranithanchai W., Karalai C., Ponglimanont C., Subhadhirasakul S., Chantrapromma K. (2009). Cassane diterpenoids from the stem of Caesalpinia pulcherrima. Phytochemistry.

[155] Roach J.S., McLean S., Reynolds W.F., Tinto W.F. (2003). Cassane Diterpenoids of Caesalpinia pulcherrima. J. Nat. Prod..

[156] Giri B.R., Bharti R.R., Roy B. (2015). In vivo anthelmintic activity of Carex baccans and its active principle resveratrol against Hymenolepis diminuta. Parasitol. Res..

[157] Li L., Henry G.E., Seeram N.P. (2009). Identification and bioactivities of resveratrol oligomers and flavonoids from carex folliculata Seeds. J. Agric. Food Chem..

[158] González-Sarrías A., Gromek S., Niesen D., Seeram N.P., Henry G.E. (2011). Resveratrol oligomers isolated from carex species inhibit growth of human colon tumorigenic cells mediated by cell cycle arrest. J. Agric. Food Chem..

[159] Zhou H.F., Xie C., Jian R., Kang J., Li Y., Zhuang C.L., Yang F., Zhang L.L., Lai L., Wu T. (2011). Biflavonoids from caper (*Capparis spinosa* L.) Fruits and their effects in inhibiting NF-kappa B activation. J. Agric. Food Chem..

[160] Woo E.R., Lee J.Y., Cho I.J., Kim S.G., Kang K.W. (2005). Amentoflavone inhibits the induction of nitric oxide synthase by inhibiting NF-κB activation in macrophages. Pharmacol. Res..

[161] Kawai T., Akira S. (2010). The role of pattern-recognition receptors in innate immunity: Update on toll-like receptors. Nat. Immunol..

[162] Lee S.J., Choi J.H., Son K.H., Chang H.W., Kang S.S., Kim H.P. (1995). Suppression of mouse lymphocyte proliferation in vitro by naturally-occurring biflavonoids. Life Sci..

[163] Lakshmi V., Joseph S.K., Srivastava S., Verma S.K., Sahoo M.K., Dube V., Mishra S.K., Murthy P.K. (2010). Antifilarial activity in vitro and in vivo of some flavonoids tested against Brugia malayi. Acta Trop..

[164] Cheng X., Ivanov I., Reisfeld B., Mayeno A. (2012). Molecular Dynamics. Computational Toxicology Methods in Molecular Biology (Methods and Protocols).

[165] Dong Y.W., Liao M.L., Meng X.L., Somero G.N. (2018). Structural flexibility and protein adaptation to temperature: Molecular dynamics analysis of malate dehydrogenases of marine molluscs. Proc. Natl. Acad. Sci. USA.

[166] Perez A., Morrone J.A., Simmerling C., Dill K.A. (2016). Advances in free-energy-based simulations of protein folding and ligand binding. Curr. Opin. Struct. Biol..

[167] Ganesan A., Coote M.L., Barakat K. (2017). Molecular dynamics-driven drug discovery: Leaping forward with confidence. Drug Discov. Today.

[168] Deng N., Zhang P., Cieplak P., Lai L. (2011). Elucidating the Energetics of Entropically Driven Protein–Ligand Association: Calculations of Absolute Binding Free Energy and Entropy. J. Phys. Chem. B.

[169] Kwofie S., Dankwa B., Enninful K., Adobor C., Broni E., Ntiamoah A., Wilson M. (2019). Molecular Docking and Dynamics Simulation Studies Predict Munc18b as a Target of Mycolactone: A Plausible Mechanism for Granule Exocytosis Impairment in Buruli Ulcer Pathogenesis. Toxins.

[170] Mathabe M.C., Hussein A.A., Nikolova R.V., Basson A.E., Meyer J.J.M., Lall N. (2008). Antibacterial activities and cytotoxicity of terpenoids isolated from Spirostachys africana. J. Ethnopharmacol..

[171] Kishore V., Puttaraju H. (2019). Homology modelling and simulation of Wolbachia surface protein (WSP) of Uzifly and study of its Insilico protein-protein anti-apoptosis interaction process with ethanol stressed HepG2 Cell line pathway proteins. J. Pharmacogn. Phytochem..

[172] Uday J., Huchesh C., Chethana V., Puttaraju H. (2014). Insilco Analysis of Wolbachia Surface Protein in Wolbachia Endosymbiont of D. Melenogaster. Biomirror.

