# Supplementary material for: Molecular Docking Simulation Studies Identifies Potential Natural Product Derived-Antiwolbachial Compounds as Filaricides against Onchocerciasis
Source: Biomedicines. 2021 Nov 13;9(11):1682. doi: 10.3390/biomedicines9111682 (PMC8615632; doi:10.3390/biomedicines9111682)
Supplement: Supplementary file 1 [file biomedicines-09-01682-s001.zip › biomedicines-1406282-SI.pdf]

1 Supplementary Tables

Table S1: The top 5 protein structure templates predicted via SWISS-MODEL.

**Template ID**	**Sequence Identity (%)**	**Sequence Similarity**	**Coverage**	**Description**
1P4T	21.57	0.29	0.63	Outer membrane protein NspA.
2MLH	16.67	0.28	0.77	Opacity protein opA60.
2MAF	16.67	0.28	0.77	Opacity protein opA60.
2X27	13.78	0.26	0.81	Outer Membrane Protein OPRG.
6QAM	12.50	0.26	0.80	Outer membrane protein AlkL

Table S2: The DOPE and GA341 Scores of the 5 models predicted using Modeller.

**MODEL**	**MOD1**	**MOD2**	**MOD3**	**MOD4**	**MOD5**
**DOPE SCORE**	-14228.31055	-14368.33691	-13027.42285	-13800.28125	-13815.10254
**GA341 SCORE**	0.00340	0.00087	0.00227	0.00143	0.00162

Table S3: The C-Scores of the 5 Models Predicted by I-TASSER

**MODELS**	ITAS1	ITAS2	ITAS3	ITAS4	ITAS5
**C-SCORE**	-2.16	-2.70	-3.07	-3.39	-3.38

Table S4: Structural Evaluation of the Robetta Generated Models using SAVES v5.0

MODELS	MODEL SCORE			
	VERIFY (%)	ERRAT	PROVE (%)	PROCHECK
ROB1	65.98	75.3304	5.7	2 E, 3 W and 3 P.
ROB2	70.95	78.97	4.7	2 E, 3 W and 3 P.
ROB3	63.90	73.2719	4.4	1 E, 5 W and 2 P.
ROB4	52.70	78.0822	3.8	4 E, 2 W and 3 P.
ROB5	63.49	77.8761	6.3	1 E, 4 W and 4 P.

Table S5: Evaluation of the top 3 WSP models generated using Robetta, I-TASSER and Modeller. Models ROB2, ITAS1 and MOD2 were generated using Robetta, I-TASSER and Modeller, respectively.

	MODEL SCORE		
	ROB2	ITAS1	MOD2
VERIFY	70.95 %	72.2 %	9.96 %
ERRAT (Quality Factor)	78.97	78.1116	35.9649
PROVE	4.7 %	10 %	9.3 %
PROCHECK	2 Errors, 3 Warnings and 3 Passes.	7 Errors, 1 Warning and 0 Pass.	3 Errors, 2 Warning and 3 Passes

Table S6: ADME Prediction of some compounds for Gastrointestinal (GI); Blood Brain Barrier (BBB); Estimated Solubility (ESOL) class, P-glycoprotein (Pgp) and TPSA.

**Compound**	**Molecular Weight (g/mol)**	**cLogP**	**ESOL Class**	**GI Absorption**	**BBB permeant**	**Pgp substrate**	**TPSA**	**No of Veber's rule Violations**	**No. of Lipinski's rule violations**
ZINC000095913861	556.6	7.6813	Poorly soluble	Low	No	No	94.56	1	0
ZINC000070455413	532.49	1.037	Soluble	Low	No	Yes	154.76	2	1
ZINC000103584225	590.53	5.5823	Poorly soluble	Low	No	Yes	155.89	1	1
ZINC000085530783	588.86	7.4895	Poorly soluble	Low	No	No	66.76	2	0
ZINC000085594065	580.63	5.4685	Poorly soluble	Low	No	No	137.41	1	0
ZINC000100822646	494.49	4.3531	Poorly soluble	Low	No	No	135.29	0	0
NANPDB5642	540.6	1.0884	Soluble	Low	No	Yes	147.43	1	1
ZINC000095486235	448.51	2.2118	Moderately soluble	High	Yes	No	71.47	0	0
NANPDB2874	433.46	2.1264	Moderately	High	No	Yes	109.05	0	0

			soluble						
ZINC000035941652	324.37	4.0376	Moderately soluble	High	Yes	Yes	55.76	0	0
NANPDB4566	382.49	4.4305	Moderately soluble	High	Yes	Yes	59.67	0	0
NANPDB513	532.62	0.7872	Soluble	High	No	Yes	131.75	1	0
Acetylaleuritolic acid	498.74	6.5489	Poorly soluble	Low	No	No	63.60	1	0
Rhemannic acid	552.78	7.1035	Poorly soluble	Low	No	Yes	80.67	2	0
Polycarpol	440.70	7.2941	Poorly soluble	Low	No	No	40.46	1	0

Table S7: Toxicological profiles of some selected compounds.

**Compound**	**Mutagenic**	**Tumorigenic**	**Reproductive Effect**	**Irritant**
ZINC000095913861	None	None	High	None
ZINC000070455413	None	None	None	None
ZINC000103584225	High	None	None	None
ZINC000085530783	None	None	None	None
ZINC000085594065	Low	Low	High	High
ZINC000100822646	None	None	None	None
NANPDB5642	None	None	None	None
ZINC000095486235	None	None	None	None
NANPDB2874	None	None	None	None
ZINC000035941652	None	None	None	None
NANPDB4566	None	None	None	None
NANPDB513	None	None	None	None
Acetylaleuritolic acid	None	None	None	None
Rhemannic acid	None	None	None	High
Polycarpol	None	None	None	High

Table S8: Binding energies of the top 20 non-redundant lead compounds each of the TCM and Afro libraries and the top 11 onchocerca spp compounds against the three top models (ROB2, ITAS1 and MOD2).

COMPOUND	SOURCE	BINDING AFFINITY		
		ROB2	ITAS1	MOD2
ZINC000095913861	TCM	-12.7	-10.2	-11.9
ZINC000085594065	TCM	-11.4	-8.2	-10.3
ZINC000100822646	TCM	-11.4	-7.8	-9.4
ZINC000103543220	TCM	-11.3	-9.3	-10.1
ZINC000002151115	TCM	-11.2	-7.8	-9.3
ZINC000042876996	TCM	-11.2	-8.0	-9.2
ZINC000004731235	TCM	-11.1	-7.5	-9.3
ZINC000085594104	TCM	-11.1	-9.0	-9.6
ZINC000085594687	TCM	-11.1	-7.4	-8.9
ZINC000095910593	TCM	-11.1	-8.7	-8.7
ZINC000095912193	TCM	-11.1	-8.1	-9.2
NANPDB2874	Afro	-11.1	-7.5	-8.5
ZINC000095486235	Afro	-11.1	-7.1	-9.4
ZINC000070454399	TCM	-11.0	-7.3	-8.8
ZINC000085530164	TCM	-11.0	-10.1	-9.2
ZINC000085543245	TCM	-11.0	-8.3	-10.2
ZINC000085568136	TCM	-11.0	-8.7	-10.1
ZINC000085568150	TCM	-11.0	-8.6	-11.5
ZINC000085594672	TCM	-11.0	-7.4	-8.4
NANPDB4566	Afro	-11.0	-7.9	-9.3
NANPDB513	Afro	-11.0	-7.5	-9.4
ZINC000035941652	Afro	-11.0	-7.7	-9.4
ZINC000013384209	TCM	-10.9	-8.0	-9.7
ZINC000070450912	TCM	-10.9	-8.5	-9.2
ZINC000070454467	TCM	-10.9	-8.7	-9.8
NANPDB4306	Afro	-10.9	-7.8	-9.3
ZINC000095485962	Afro	-10.9	-8.8	-10.3
NANPDB1649	Afro	-10.7	-7.5	-9.2
NANPDB3795	Afro	-10.7	-7.7	-10.4
NANPDB4605	Afro	-10.7	-7.7	-9.5
NANPDB2966	Afro	-10.6	-7.4	-10.1
NANPDB4136	Afro	-10.6	-7.3	-8.6
ZINC000100003095	Afro	-10.6	-8.8	-9.3
NANPDB347	Afro	-10.5	-7.5	-9.1
NANPDB4312	Afro	-10.5	-8.0	-9.2
ZINC000095485942	Afro	-10.5	-8.8	-9.4
ZINC000095486126	Afro	-10.5	-7.9	-9.6
NANPDB488	Afro	-10.4	-7.6	-9.2
ZINC000095485936	Afro	-10.4	-7.7	-9.2
ZINC000095486074	Afro	-10.4	-8.1	-9.2
Acetylaleuritolic acid	Oncho	-10.3	-7.9	-9.1
Rhemannic acid	Oncho	-9.5	-8.4	-8.9
Polycarpol	Oncho	-9.2	-8.2	-8.8

Ellagic acid	Oncho	-8.6	-6.8	-8.2
Voacangine	Oncho	-7.9	-6.6	-7.1
Germacrone	Oncho	-7.5	-7.1	-6.6
Cardinol	Oncho	-7.4	-7.2	-6.5
Cyperotundone	Oncho	-7.4	-6.7	-6.7
Alpha terpinyl acetate	Oncho	-7.2	-6.3	-5.8
Caryophyllene oxide	Oncho	-7.2	-6.7	-6.7
Cedrol	Oncho	-7.0	-7.1	-6.7

Table S9: Previously modelled WSP structures and the templates used. N/A: Not available in article.

Organism WSP is found	WSP Sequence ID	Template for Modelling	Sequence Identity of Template (%)	Type of Study	Reference
Drosophila melanogaster	NP_966785.1 (SWISSPROT)	Chain A: Crystal Structure of Neisserial Surface Protein A (Nspa) PDB ID: 1P4T	34	1. Modelling of WSP in *D. melanogaster* [1].<br><br>2. Comparative study of 3 different WSP modelled structures (in *D. melanogaster*, *A. tabida* and *B. malayi*) [2].	[1,2]
Asobara tabida	AAW78819.1 (SWISSPROT)	Chain A: Crystal Structure of Neisserial Surface Protein A (Nspa) PDB ID: 1P4T	N/A		[2]
Brugia malayi	AAW70873.1 (SWISSPROT)	Chain A: Crystal Structure of Neisserial Surface Protein A (Nspa) [PDB ID: 1P4T]	N/A		[2]
Exorista sorbillans	G1EHU4 (UniProt)	1. Chain B: Stonustoxin Structure (PDB ID: 4WVM)<br><br>2. Chain A: Hemagglutinin-esterase-fusion Mutant Structure of Influenza D Virus (PDB ID: 5E5W)<br><br>3. Chain A: Crystal Structure of Neisserial Surface Protein A (Nspa) [PDB ID: 1P4T]<br><br>4. Chain A:	30<br><br>25<br><br>27<br><br>24	The study modelled the WSP structure using multiple-templates homology modelling (using all 5 templates) [3]. The study also modelled 5 different structures using each of the 5 templates. The structures were then compared and the 1P4T-based WSP structure was selected as the reasonably best upon evaluating the quality of the models [3].<br>The selected structure was docked against 7	[3]

		Hemagglutinin-esterase-fusion Protein Structure of Influenza D Virus (PDB ID: 5E64)<br><br>5. Chain A: NMR Solution Structure of Opa60 from *N. gonorrhoeae* (PDB ID: 2MLH)	57	proteins involved in the Ethanol stressed pathway of HepG2 cells [3].	

2 Supplementary Figures

A)

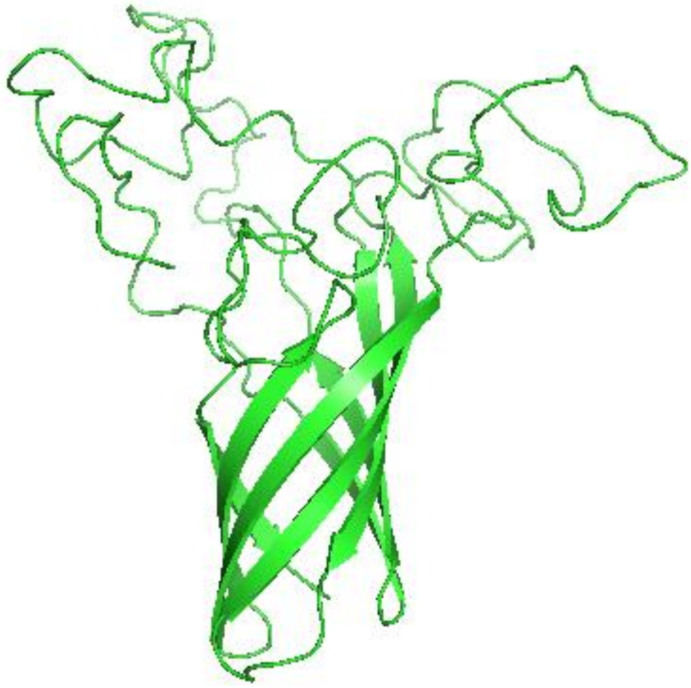

B)

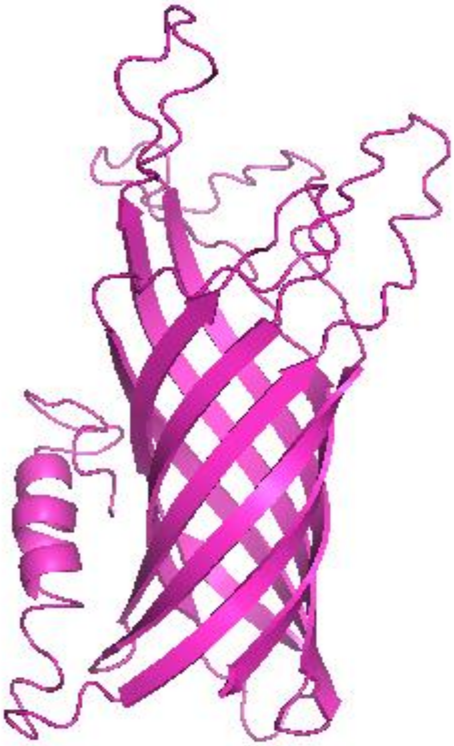

C)

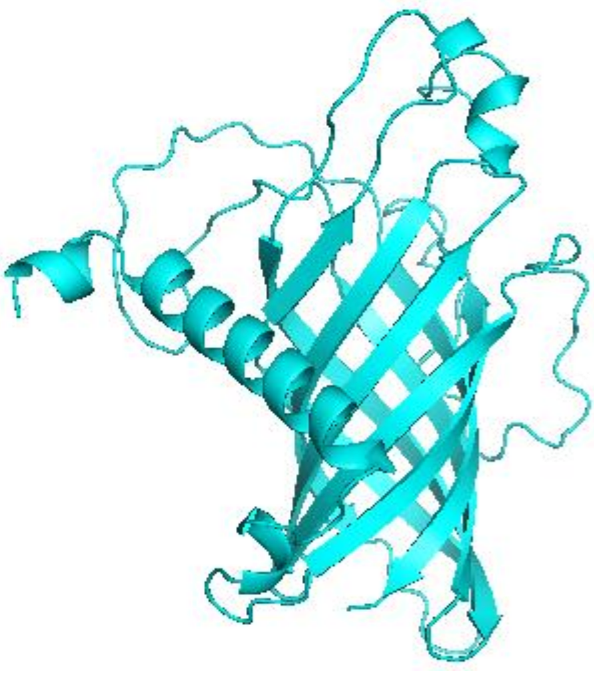

Figure S1: Cartoon representation of the top 3 predicted tertiary structures of the *Wolbachia* surface protein from the 3 techniques used: (A) MOD2 (B) ITAS1 and (C) ROB2. ITAS1 is in magenta, ROB2 in cyan and MOD2 in green.

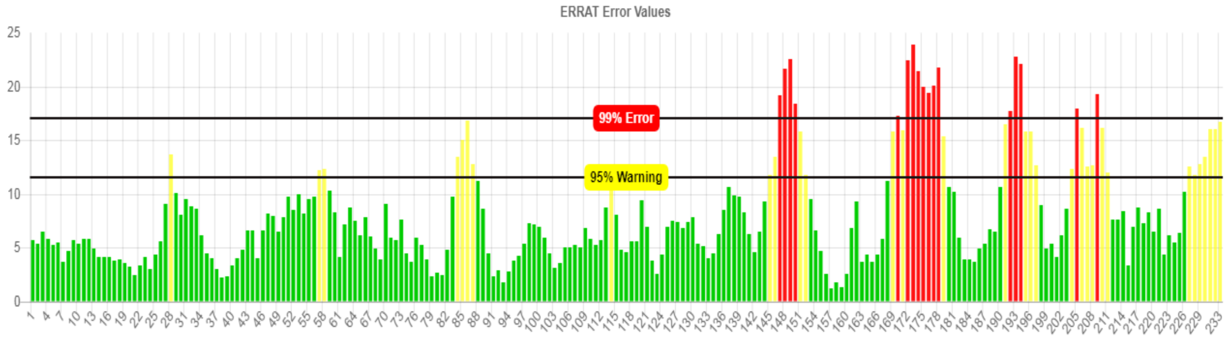

Figure S2: ERRAT plot for the WSP model. Red bars identify the misfolded region located distantly from the active site, yellow bars demonstrate the error region between 95% and 99%, and green bars indicate the region with a lower error rate for protein folding.

A)

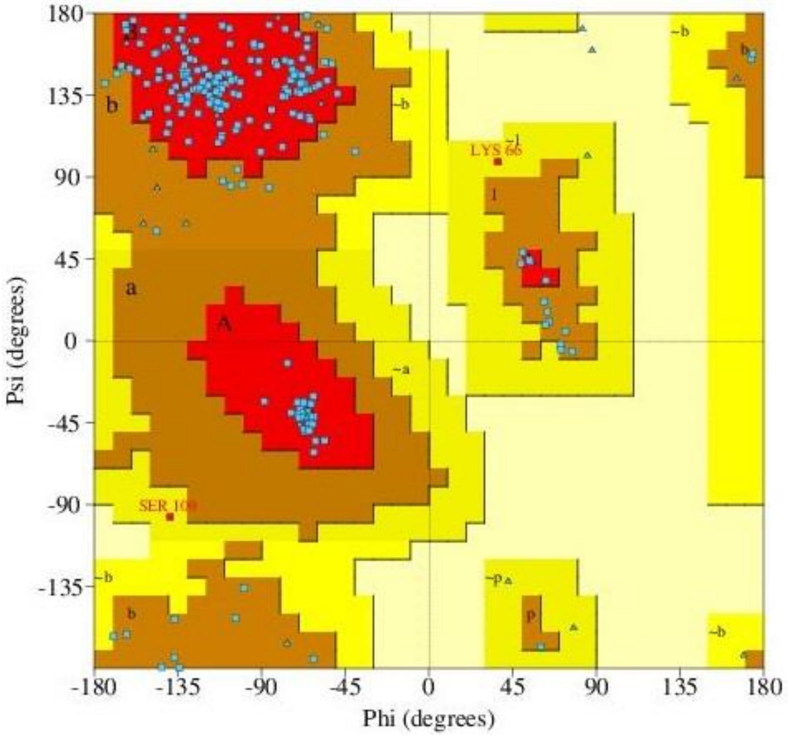

B)

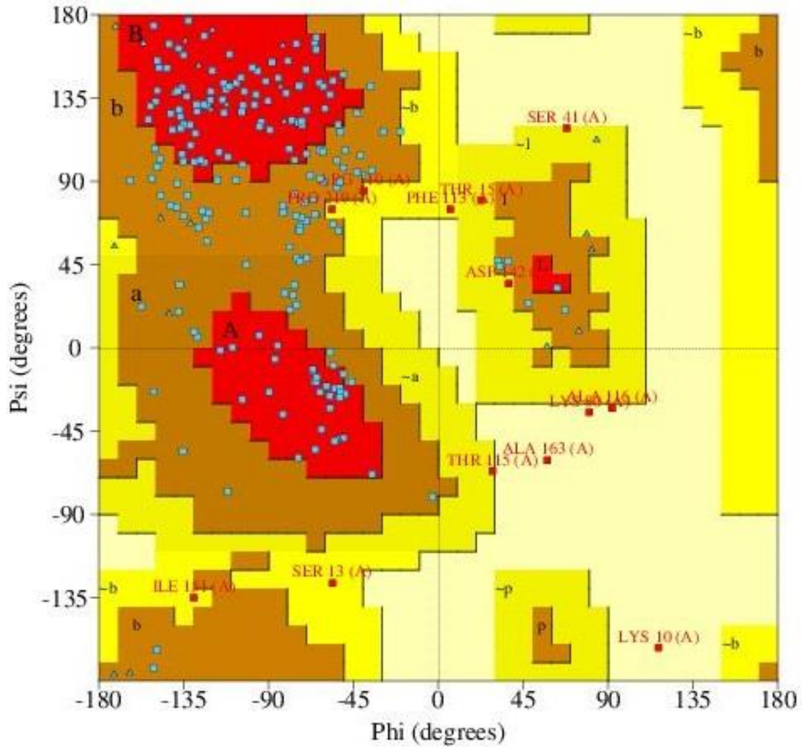

Figure S3: Ramachandran plots of the protein structures obtained via PROCHECK. (A) Ramachandran plot of protein model MOD2 and (B) Ramachandran plot of protein model

ITAS1. The percentages of residues of model MOD2 in the most favoured regions, additionally allowed regions, generously allowed regions and disallowed regions are 82.5, 16.5, 1.0 and 0.0%, respectively. For model ITAS1, 53.9, 40.3, 3.9 and 1.9% of the amino acid residues were predicted to be in the most favoured, additionally allowed, generously allowed and disallowed regions, respectively.

A)

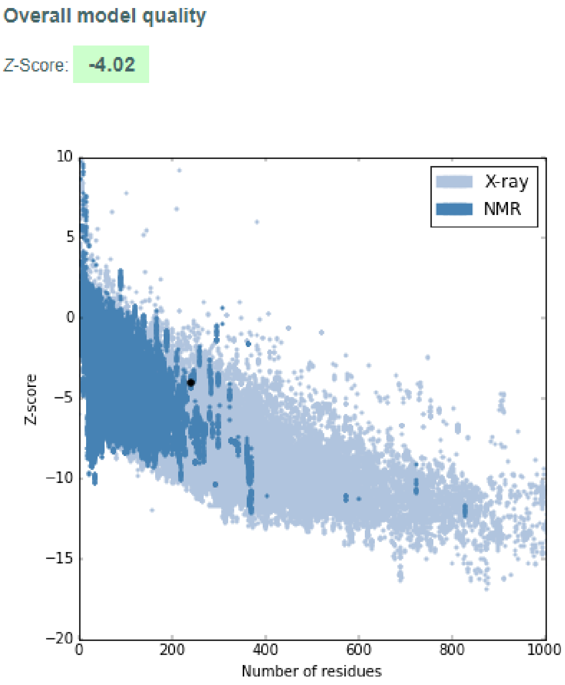

B)

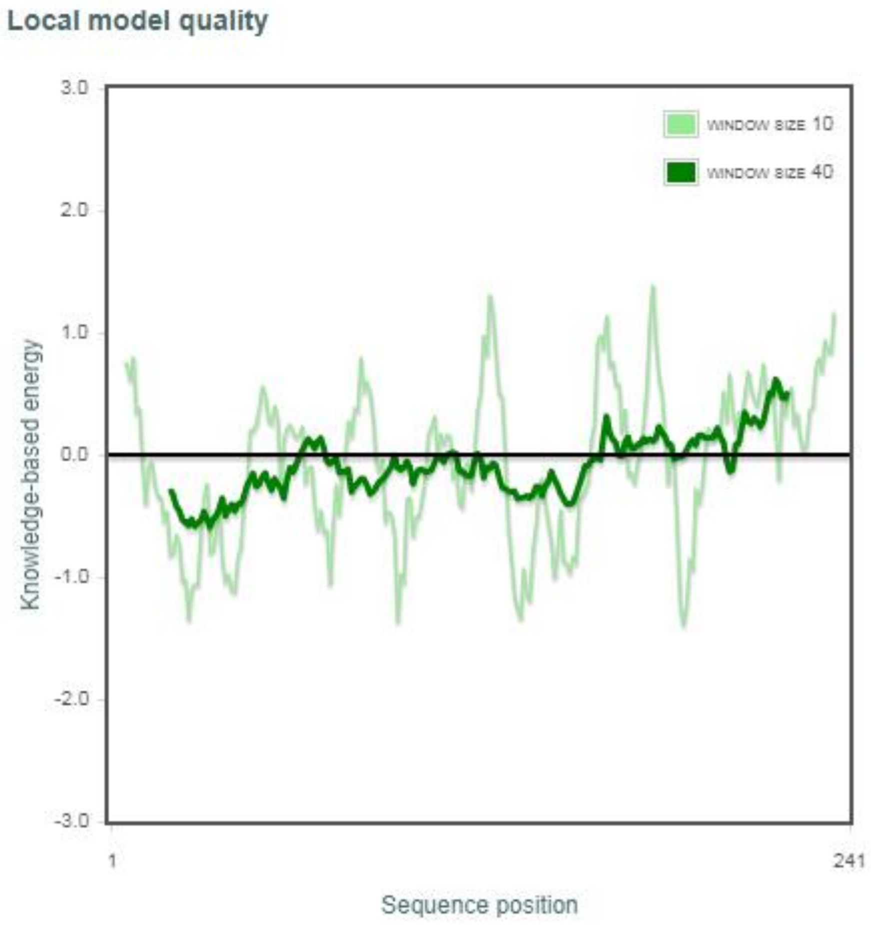

Figure S4: Protein quality evaluation using ProSA. A) ProSA-web z-score of the WSP indicating the overall model quality. B) ProSA-web local model quality of the WSP by plotting energies as a function of amino acid sequence position.

A）

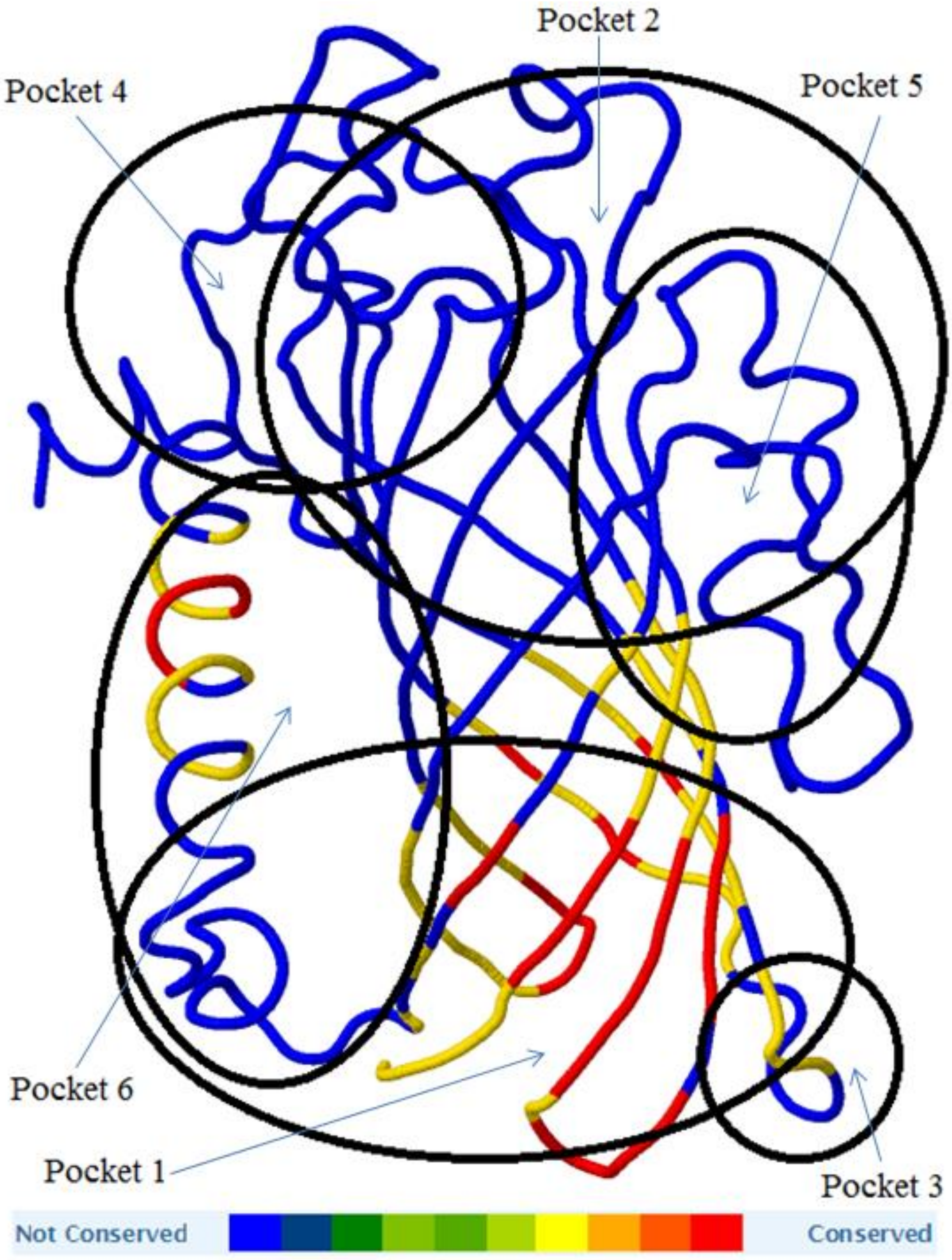
Pocket 2
Pocket 4
Pocket 5
Pocket 6
Pocket 1
Pocket 3
Not Conserved
Conserved

B

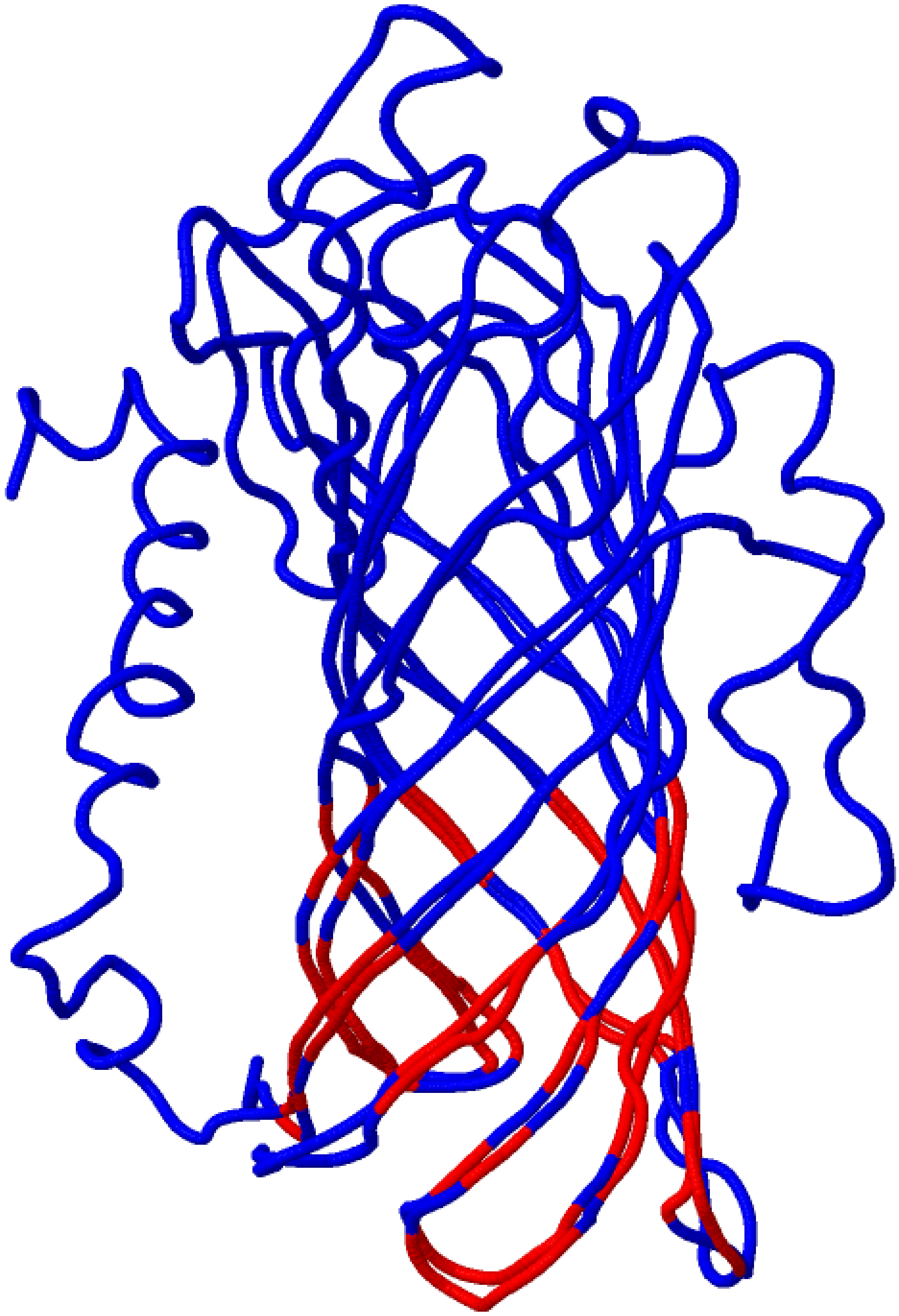

C)

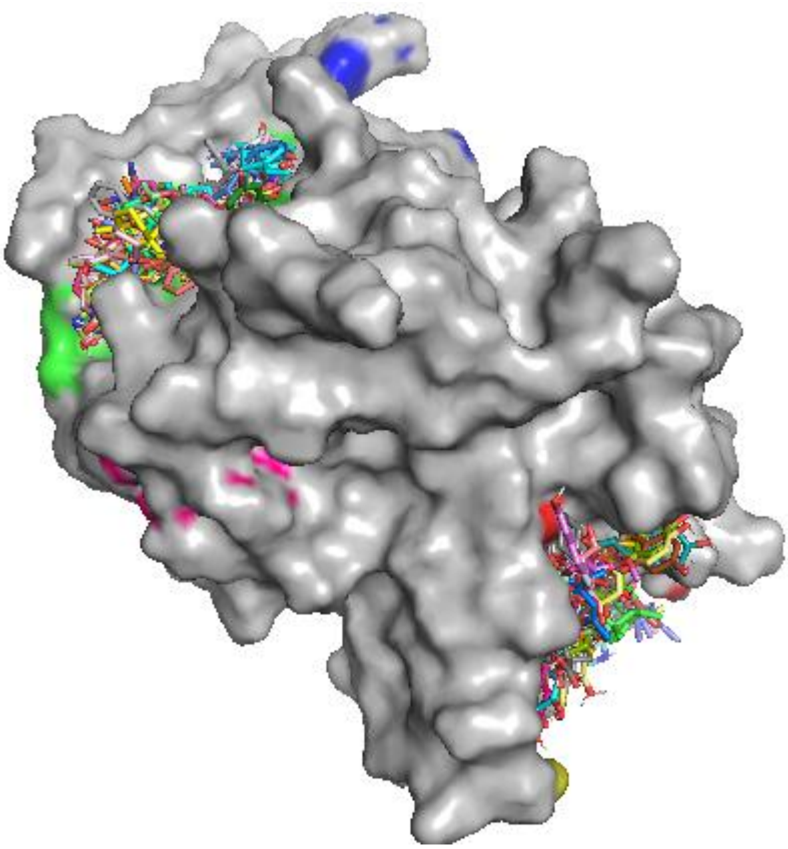

Figure S5: Predicted binding sites of the WSP (ROB2 model). (A) Binding site positions are shown with respect to conservation; (B) Superimposition of WSP (ROB2 model) and 1P4T (template structure) showing conserved binding site region in red and unconserved sites in blue; and (C) Ligands docked into the binding sites of the WSP. In (C), the protein is shown as a grey surface representation while pockets 1, 2, 3, 4, 5 and 6 are coloured red, green, yellow, blue, hotpink and cyan, respectively.

A)

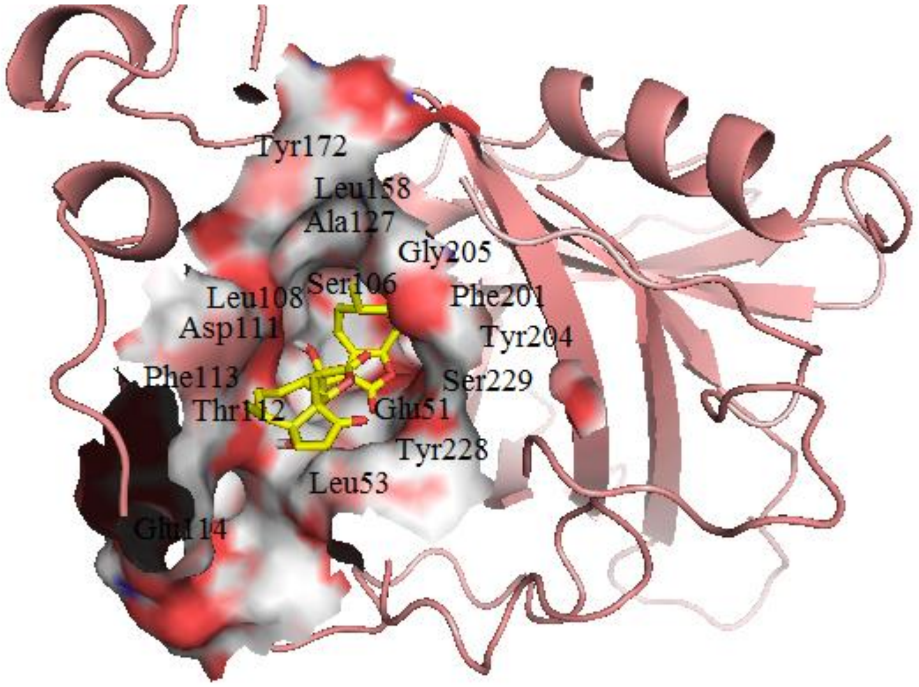
Tyr172
Leu158
Ala127
Gly205
Ser106
Leu108
Phe201
Asp111
Tyr204
Phe113
Ser229
Thr112
Glu51
Tyr228
Leu53
Glu114

B)

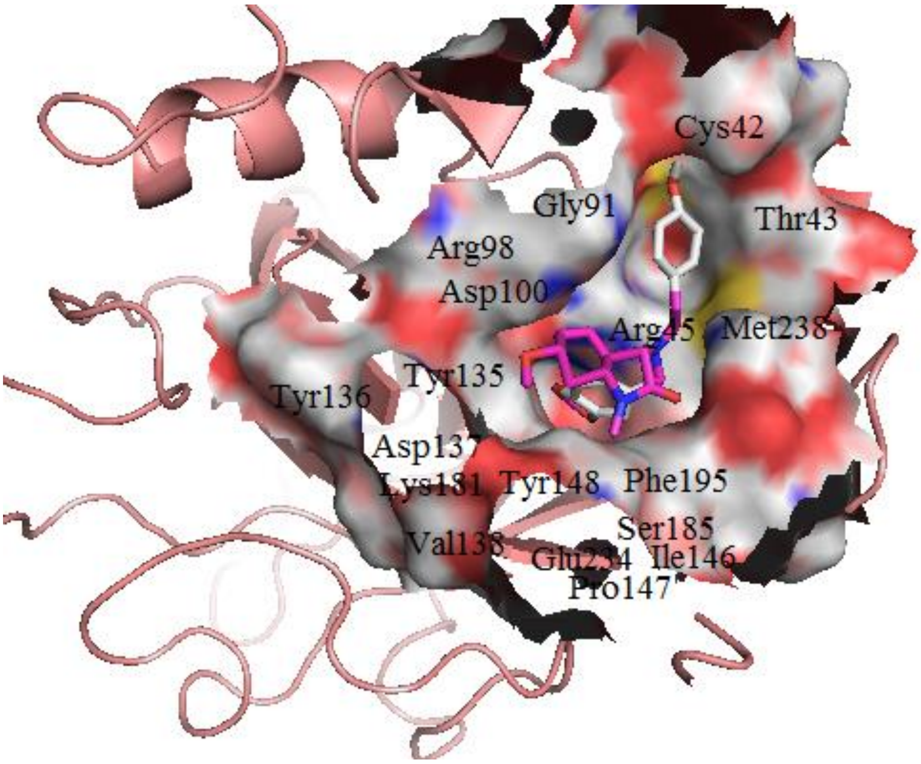
Cys42
Gly91
Thr43
Arg98
Asp100
Arg45
Met238
Tyr135
Tyr136
Asp137
Lys181
Tyr148
Phe195
Ser185
Val138
Glu234
Ile146
Pro147

C)

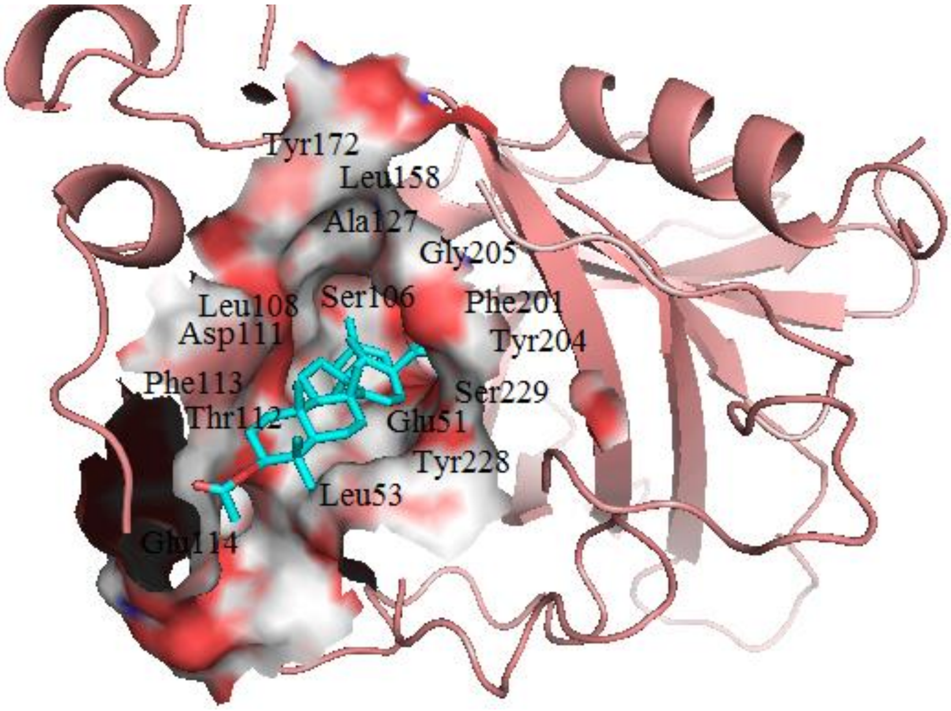

Figure S6: Cartoon representation of the WSP in complex with (A) NANPDB5642, (B) ZINC000095486235 and (C) acetylaleuritolic acid. The binding sites are represented as surface and the ligands are shown as sticks.

A)

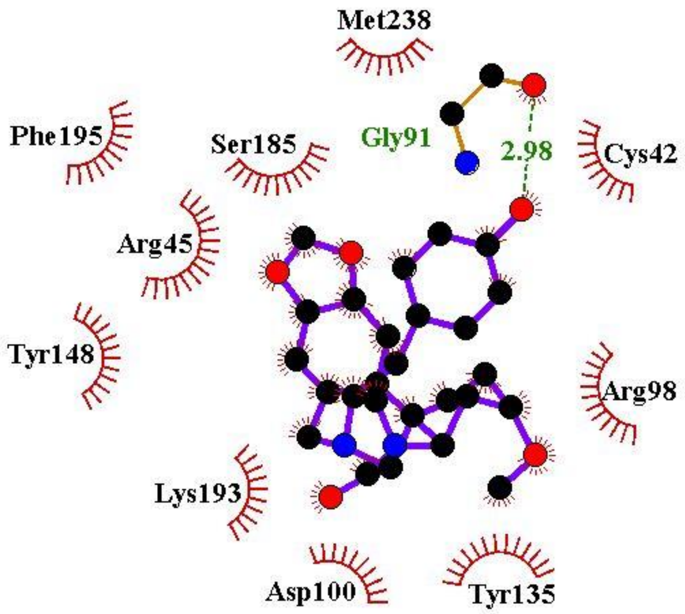

B)

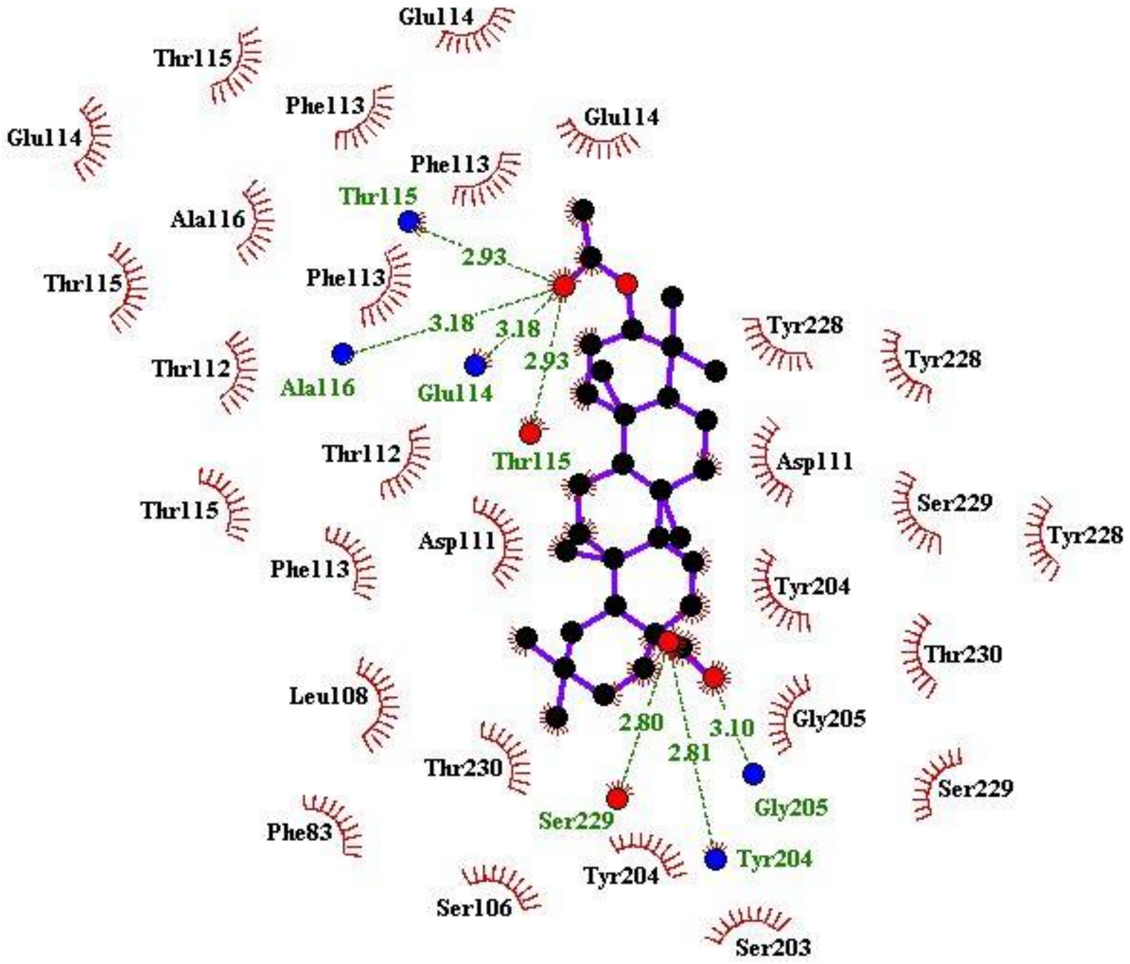

Figure S7: Two-dimensional plots of the WSP-ligand interactions generated using LigPlot+. Interaction profiles of (A) WSP- ZINC000095486235 complex and (B) WSP-Acetylaleuritolic acid complex. Hydrophobic contacts are represented as red spoke arcs, hydrogen bonds are represented as green dash lines and the ligands are colored purple.

A)

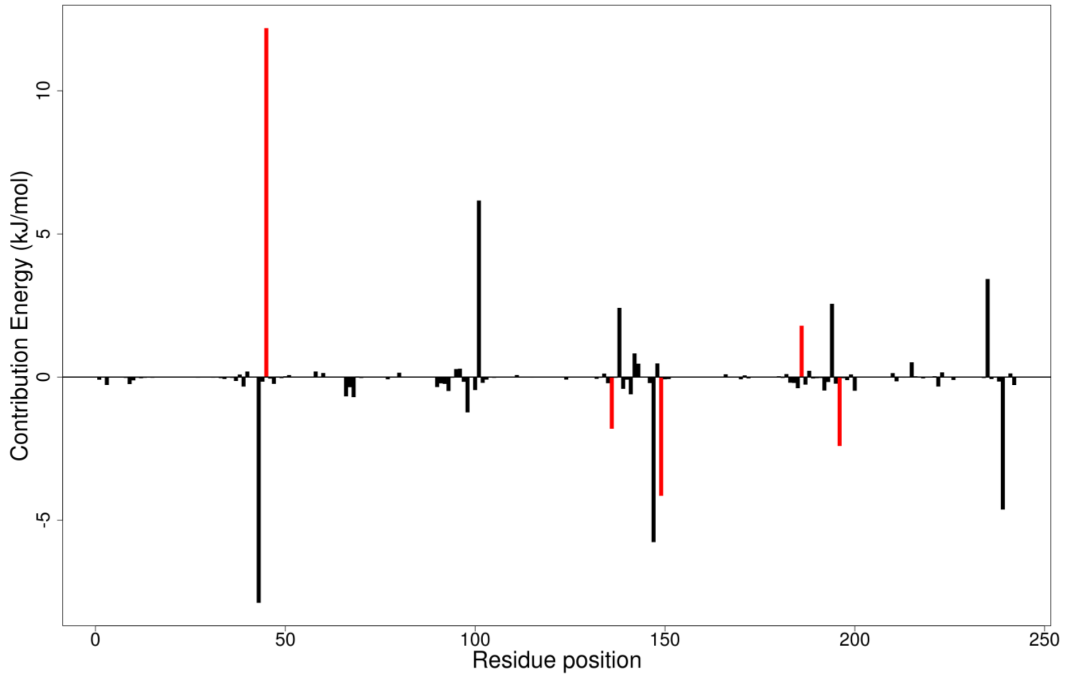

Contribution Energy (kJ/mol)
10
5
0
-5
0
50
100
150
200
250
Residue position

B)

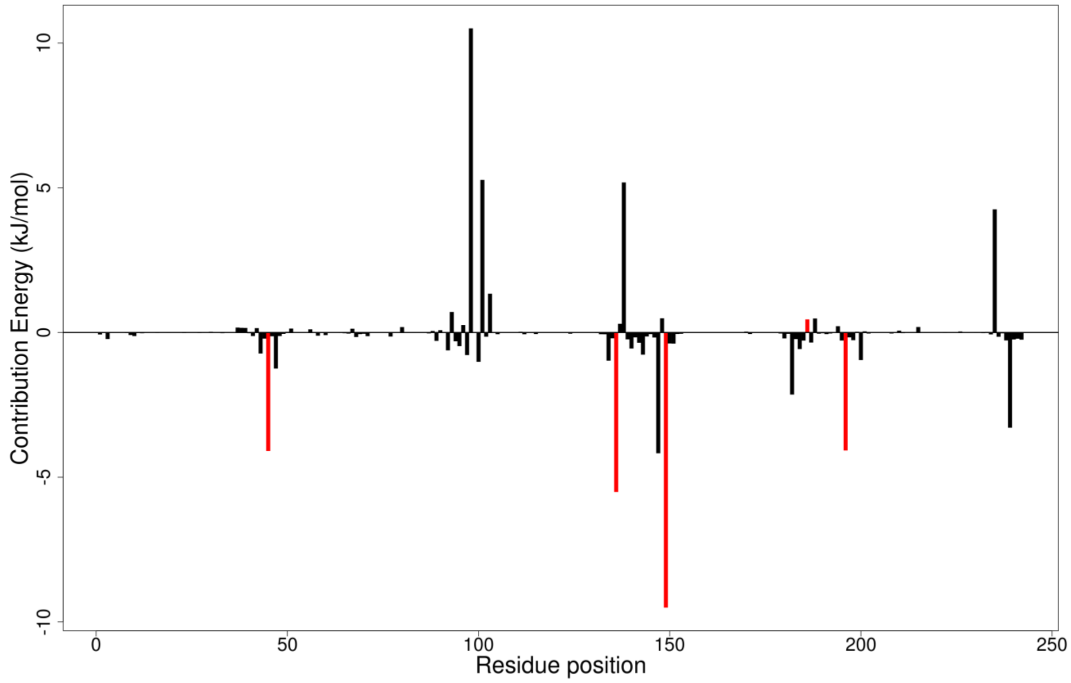

Contribution Energy (kJ/mol)
10
5
0
-5
-10
0
50
100
150
200
250
Residue position

C)

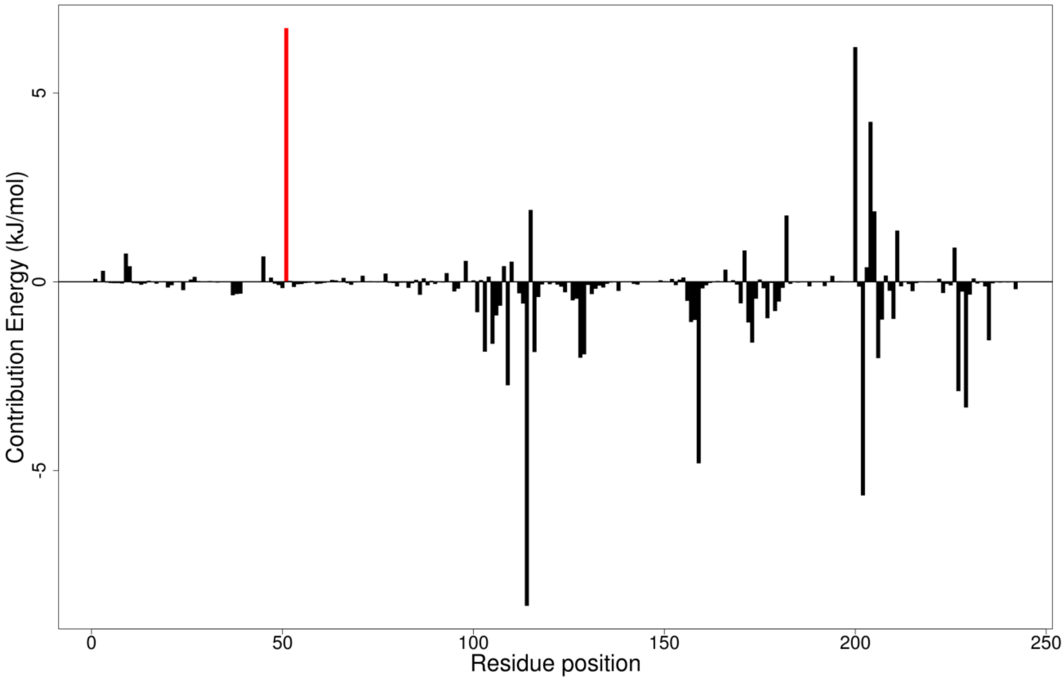

Contribution Energy (kJ/mol)
5
0
-5
0
50
100
150
200
250
Residue position

D)

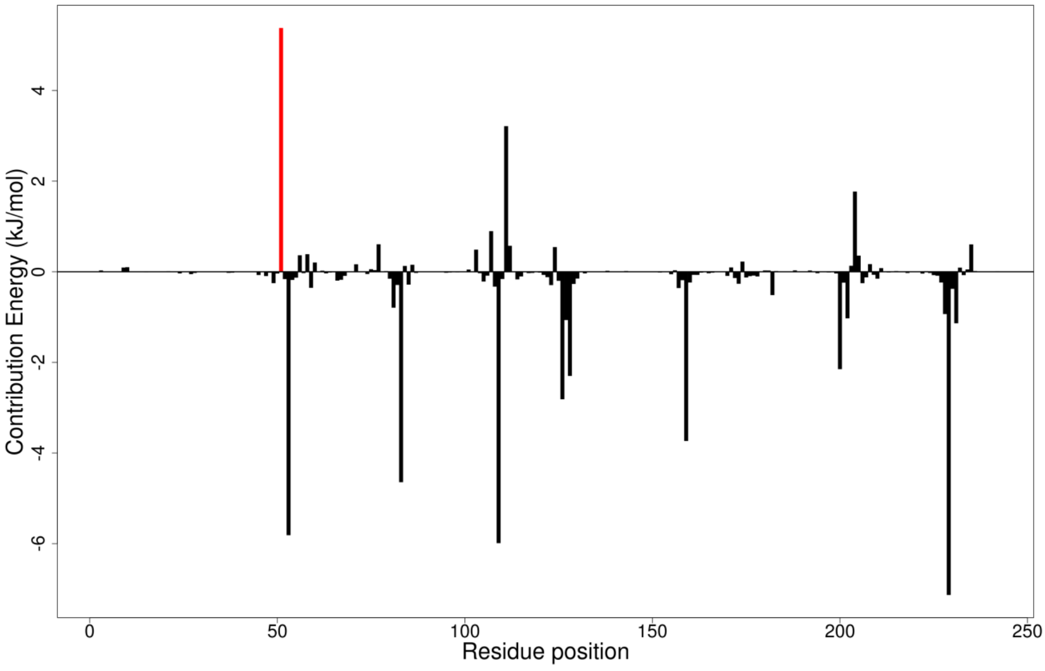

Contribution Energy (kJ/mol)
4
2
0
-2
-4
-6
0
50
100
150
200
250
Residue position

E)

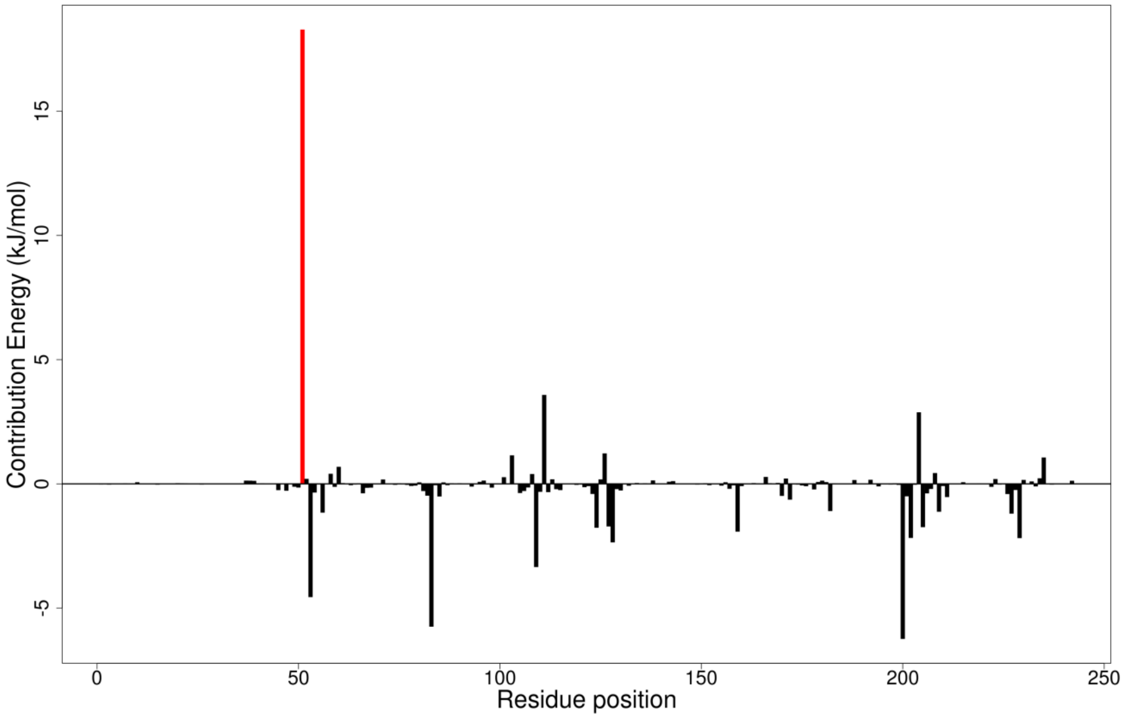

Figure S8: Molecular mechanics/Poisson-Boltzmann surface area (MMPBSA) plots showing the binding free energy contribution per residue of the (A) WSP-ZINC000095486235, (B) WSP-ZINC000035941652, (C) WSP-ZINC000095913861, (D) WSP-acetylaleuritolic acid and (E) WSP-rhemannic acid complexes.

3 References

1. Uday, J.; Huchesh, C.; Chethana, V.; Puttaraju, H. Insilco Analysis of Wolbachia Surface Protein in Wolbachia Endosymbiont of D. Melenogaster. *Biomirror* **2014**, *5*, 24–29.

2. Uday, J.; Puttaraju, H.P. Comparative analysis of Wolbachia surface protein in D. melanoagster, A. tabida and B. malayi. *Bioinformation* **2012**, *8*, 711–715, doi:10.6026/97320630008711.

3. Kishore, V.; Puttaraju, H. Homology modelling and simulation of Wolbachia surface protein (WSP) of Uzifly and study of its Insilico protein-protein anti-apoptosis interaction process with ethanol stressed HepG2 Cell line pathway proteins. *J. Pharmacogn. Phytochem.* **2019**, *8*, 618–624.
